# Assessment of the fish tumors or other deformities beneficial use impairment and associated risks at two Lake Michigan Areas of Concern

**DOI:** 10.1007/s10646-025-03001-8

**Published:** 2026-01-03

**Authors:** V. S. Blazer, C. R. Smith, H. L. Walsh, P. M. Mazik, M. R. Magee

**Affiliations:** 1 Eastern Ecological Science Center-Leetown Research Laboratory, U.S. Geological Survey, Kearneysville, WV USA; 2 West Virginia Cooperative Fish and Wildlife Unit, West Virginia University, Morgantown, WV USA; 3 Wisconsin Department of Natural Resources, Madison, WI USA

**Keywords:** White sucker, Liver neoplasms, Skin neoplasms, Carcinogenesis

## Abstract

The U.S. Great Lakes Areas of Concern (AOCs) have been designated due to significant environmental degradation, with multiple Beneficial Use Impairments (BUIs) requiring remediation before delisting. The “fish tumors or other deformities” BUI remains at the Sheboygan River and the Lower Green Bay and Fox River AOCs, necessitating evaluation in white sucker (*Catostomus commersonii*), a sentinel species. This study documented the prevalence of skin and liver neoplastic and preneoplastic lesions at these AOCs, assessed temporal trends at the Sheboygan River AOC from 2012 to 2021, and investigated potential risk factors influencing liver neoplasm development using histological and molecular approaches. Histopathological analyses revealed that skin neoplasms were present in 15.5% of fish from the Green Bay AOC and 21.5% from the Sheboygan River AOC, while liver neoplasms were detected in 7.0% and 6.0% of fish, respectively. At the Sheboygan River AOC, the prevalence of skin tumors declined from 32.6% in 2012 to 21.5% in 2021, and liver tumor prevalence decreased from 8.3% in 2012 to 6.0% in 2021. Hepatic transcript analyses identified significant expression of genes associated with contaminant exposure, oxidative stress, and immune response, suggesting ongoing exposure to environmental stressors. The presence of white sucker hepatitis B-like virus (WSHBV) in hepatocytes was confirmed, though no direct correlation between viral transcript abundance and neoplastic lesions was found. Findings indicate tumor prevalence has declined over time, however rates remain elevated compared to previous reference site assessments. The study underscores the potential multifactorial nature of carcinogenesis in wild fish populations, involving both chemical and biological stressors.

**Supplementary Information:**

The online version contains supplementary material available at 10.1007/s10646-025-03001-8.

## Introduction

The Great Lakes have been significantly impacted by anthropogenic activities, leading to the designation of Areas of Concern (AOCs), defined as geographic regions where environmental degradation as a result of local human activities has impaired ecosystem health and beneficial uses. Under the U.S.-Canada Great Lakes Water Quality Agreement (Annex 1 of the 2012 Protocol), AOCs require remediation and monitoring to restore beneficial uses. Currently, 25 U.S. AOCs remain, where restoration and monitoring of Beneficial Use Impairments (BUIs) is occurring (www.epa.gov/great-lakes-aocs*).* Among these, the Sheboygan River and the Lower Green Bay and Fox River (subsequently referred to as Green Bay) AOCs, both located within the Lake Michigan watershed, continue to exhibit BUIs, including “fish tumors or other deformities.” This impairment is characterized by elevated incidence rates of neoplastic or preneoplastic liver tumors in indicator species such as bullheads and suckers, exceeding those observed at unimpacted control sites. Understanding the prevalence and risk factors for this impairment can help to assess ecosystem recovery and inform future remediation efforts.

The Green Bay AOC is contaminated with chemicals such as polychlorinated biphenyls (PCBs), polycyclic aromatic hydrocarbons (PAHs), and metals (www.epa.gov/great-lakes-aocs/lower-green-bayfox-river-aoc). In 2013, actions necessary for restoring and delisting the remaining 10 Beneficial Use Impairments (BUIs) were identified, and by 2020, all sediment remediation projects were completed. Monitoring efforts were initiated to evaluate the remaining BUIs, including the “fish tumors and other deformities”, which had not been recently assessed. Similarly, the Sheboygan River AOC was created in 1987 due to contamination from industrial wastes, sewage treatment plants, illegal dumping, improper hazardous waste disposal, and nonpoint pollution sources (www.epa.gov/great-lakes-aocs/sheboygan-river-aoc/sheboygan). Habitat restoration and sediment remediation were completed in 2013, and the Remedial Action Plan Update (2020) outlined necessary monitoring for BUI delisting. During an initial study at the Sheboygan AOC in 1994, 16 white sucker *Catostomus commersonii* were collected for histological analyses revealing bile duct proliferation, foci of cellular alteration and one hepatic adenoma (Schrank et al. 1997). In 2012 and 2017, larger sample sizes (193 and 200 respectively) were used to assess the fish tumor BUI more accurately (Blazer et al. 2017, 2019).

White sucker were selected as the indicator species at these AOCs as they are widespread, recommended as an environmental sentinel (Munkittrick and Dixon 1989), and have been used at other United States and Canadian AOCs (Hayes et al. 1990; Ridgway et al. 1999; Bowron et al. 2009; Simmons et al. 2012; Blazer et al. 2014, 2017). The association of neoplasia with contaminant exposure in wild fishes, particularly liver tumors, has been recognized for many years (Malins et al. 1987; Myers et al. 1987; Vogelbein et al. 1990; Baumann et al. 1996; Stentiford et al. 2005). There are also numerous reports documenting higher skin neoplasm prevalence at contaminated sites (Hayes et al. 1990; Baumann et al. 1996; LeBlanc and Bain 1997). However, carcinogenesis is increasingly recognized as a multifactorial process influenced by initiators, co-carcinogens and promoters (Carbone and Pass 2004; Haverkos 2004). Consequently, as AOCs are remediated and legacy contaminant concentrations reduced, it is important to identify additional risk factors for carcinogenesis.

Evidence suggests that pathogens, including viruses or parasites may act as co-factors with chemical contaminants (legacy and emerging) in initiation and/or promotion during carcinogenesis (Baines et al. 2021; Hatta et al. 2021). During the development of the white sucker hepatic transcriptome, a notable discovery was the identification of a novel hepatitis B-like virus (WSHBV), the first hepadnavirus identified in fish (Hahn et al. 2015). In humans, hepatitis B and C are major risk factors for hepatocellular carcinoma (Ringehan et al. 2017; Alqahtani and Colombo 2020). Consequently, it is possible that viruses or other infectious agents are co-factors in both skin and liver neoplasms of white sucker.

The objectives of this study were (1) to document the most recent incidence of skin and liver neoplastic and preneoplastic lesions at the Sheboygan and Green Bay AOCs for delisting purposes; (2) compare temporal changes (2012–2021) in neoplastic and preneoplastic lesions at the Sheboygan AOC; and (3) investigate potential risk factors that may influence initiation and/or progression of liver neoplasms using microscopic and molecular techniques.

## Methods

### Field methods

**Fig. 1:**
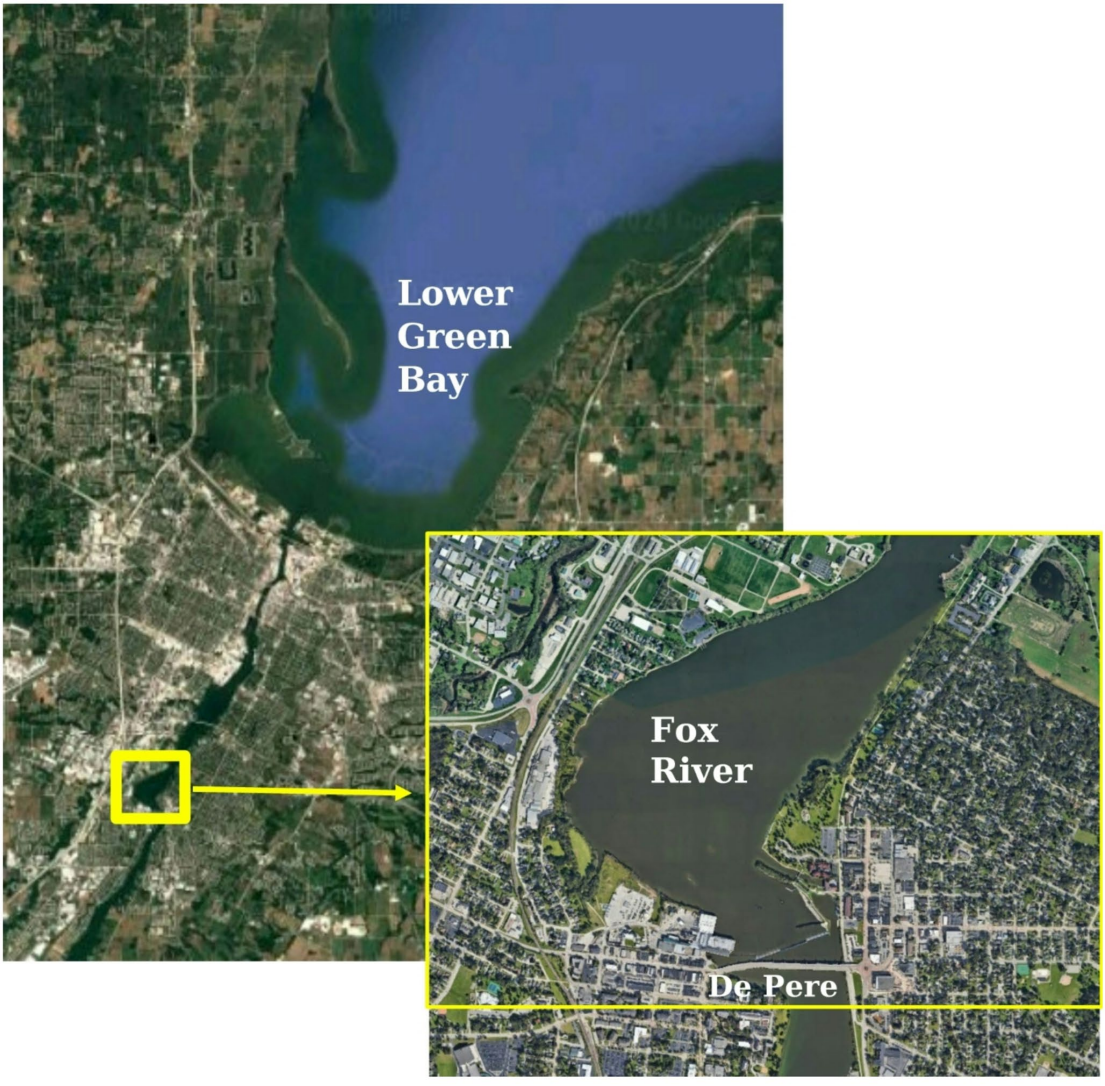
Sampling location within the Lower Green Bay/Fox River Area of Concern was downstream of the De Pere Dam (insert) in the Fox River

**Fig. 2:**
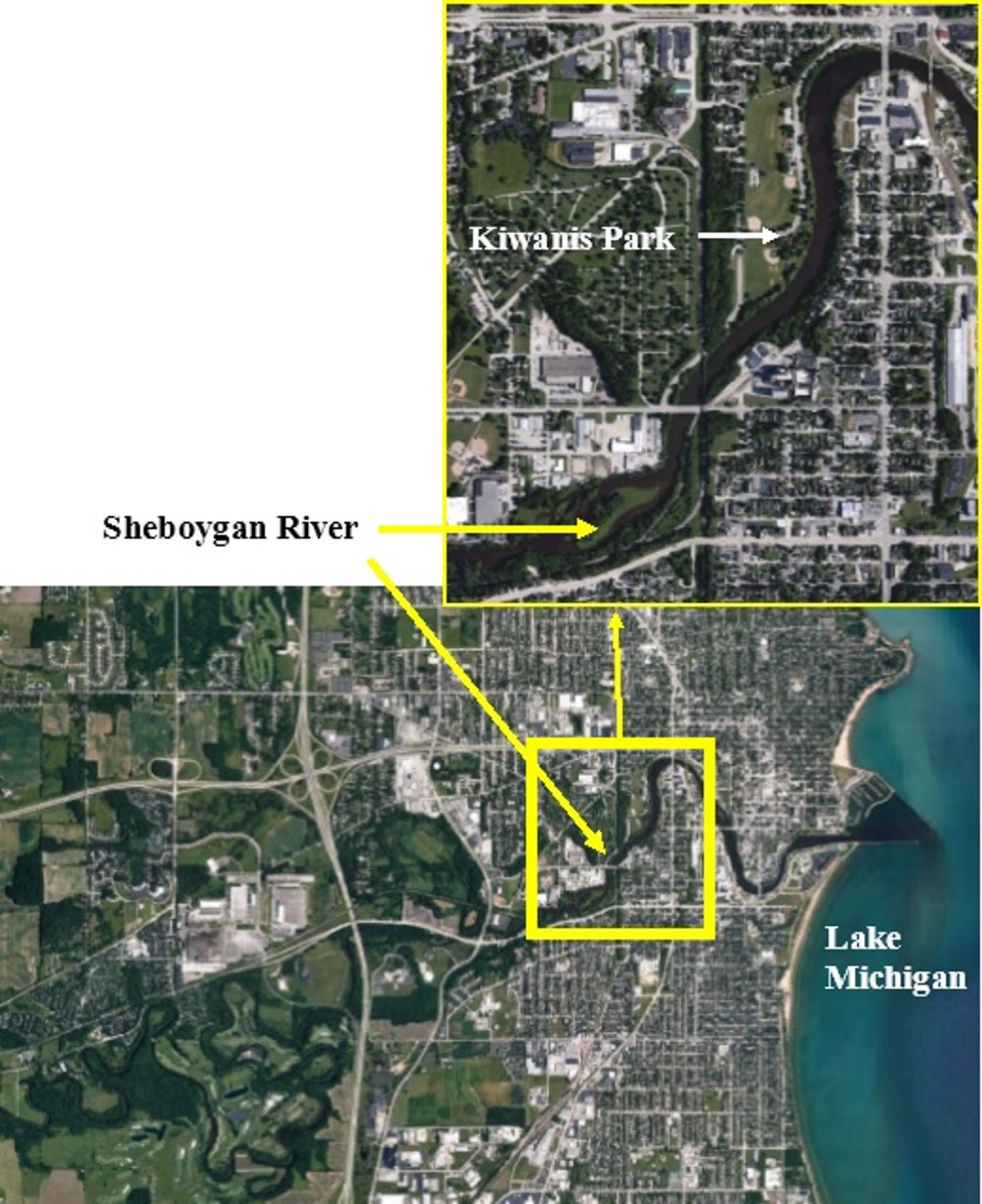
Sampling location within the Sheboygan River Area of Concern in the lower Sheboygan River upstream of Kiwanis Park (insert)

The Green Bay AOC includes the lower seven miles of the Fox River, downstream of the De Pere Dam and 22 square miles of the southern portion of Green Bay (Fig. 1). In April 2021, 200 adult white sucker were collected in the Fox River downstream of the De Pere Dam during their spawning migration. The Sheboygan River AOC includes the lower 14 miles of the Sheboygan River (downstream of the Sheboygan Falls Dam), the Sheboygan Harbor and nearshore waters of Lake Michigan (Fig. 2). In April 2021, 200 white sucker were collected during their spawning migration in the vicinity of Kiwanis Park. Fish were collected by Wisconsin Department of Natural Resources (WI DNR) personnel using boat electroshocking and held in live nets until processed (less than 24 h). Individual fish were euthanized according to the U.S. Geological Survey’s Institutional Animal Care protocol (LSC IACUC #2021-18) with tricaine methane-sulfonate (MS22, 350 mg/L mixed into river water; Western Chemical Incorporated, Ferndale, Washington), weighed and measured. External abnormalities were documented and preserved in Z-Fix™ (Anatech LtD, Battle Creek, MI) for histopathology and/or in RNAlater™ Stabilizer Solution (Thermo Fisher Scientific, Rockville, MD) for molecular analyses. Fish were necropsied and any internal abnormalities documented. At least five pieces (13–22 mm in length) were taken from different areas of the diffuse liver and preserved in Z-fix for histopathological analyses. Three to four small Sects. (3–5 mm) of liver were preserved in RNAlater™ for molecular analyses. Sex was identified by visual examination and verified by histology. Otoliths were removed for age analyses.

### Laboratory methods

Lapillus otoliths were prepared for aging with a modification (Blazer et al. 2019) of the multiple-stage process described by Koch and Quist (2007). Tissues for microscopic pathology were routinely processed through graded alcohols and embedded into paraffin. Blocks were sectioned at 5 μm and stained with hematoxylin and eosin (Luna 1992). External lesions were classified as hyperplastic or benign (papilloma) or malignant (squamous cell carcinoma) neoplasm as previously described (Smith et al. 1989; Blazer et al. 2017, 2019). Five to six sections of liver were examined and abnormalities including bile duct proliferation, foci of cellular alteration and neoplasms of the hepatic tissue (hepatic adenoma, hepatic cell carcinoma) and bile ducts (cholangioma, cholangiocarcinoma) according to previously described diagnostic criteria in fishes (Boorman et al. 1997; Blazer et al. 2006, 2017) were documented. Histology slides were independently examined by two fish pathologists (Blazer, Walsh) and consensus reached on diagnoses.

Pieces of liver preserved in RNAlater were thawed on ice and 15–25 mg of tissue extracted for total RNA with the E.Z.N.A. Total RNA Kit I (Omega Bio-Tek, Norcross, Georgia). DNA contamination was removed with a DNase treatment step (RNase-free DNase set I, Omega Bio-Tek) and samples were eluted with 50 µL nuclease-free water. An RNA BR Assay Kit (Agilent, Santa Clara, California) was used to quantify purified RNA on a Qubit 4 Fluorometer. Transcript abundance was measured using 50 ng of RNA/sample on the nCounter Max (Nanostring Technologies, Inc., Seattle, Washington), a multiplex nucleic acid hybridization technology for gene expression. A codeset of target specific sequences for white sucker as previously described by Hahn et al. (2016), modified by the addition of WSHBV sequences (Supplementary Table 1), was used. The WSHBV genome was sequenced (https://www.ncbi.gov/nuccore/NC_027922.1) and sequences included in the codeset were wshbv, a pre-genomic RNA sequence, wshbvp a and wshbvp b RNA of the polymerase gene, and wshbvs which overlaps the surface or envelope sequence and polymerase genes (Adams et al. 2021). Hepatic gene transcripts associated with stress, oxidative stress, cell proliferation, contaminant and lipid metabolism, and immune function were included (Table 1; Supplementary Table 1). Internal positive and negative controls and housekeeping transcripts were included for normalization which was conducted in nSolver 4.0 (Nansotring Technologies, Inc.).

**Table 1:** Gene transcripts measured in liver tissue of white sucker *Catostomus commersonii* collected at the Green Bay and Sheboygan river Areas of Concern

Gene Symbol	Gene Name	Related Function
*ar*	androgen receptor	reproduction-associated
*erα*	estrogen receptor α	reproduction-associated
*erβ*	estrogen receptor β	reproduction-associated
*erb2*	estrogen receptor β2 (Er-δ)	reproduction-associated
*vtg*	vitellogenin	reproduction-associated
*ahr*	aryl hydrocarbon receptor	contaminant-related
*cyp1a1*	cytochrome P450 protein	contaminant-related
*mt*	metallothionein	contaminant-related
*mt2*	metallothionein 2	contaminant-related
*gst*	glutathionine S-transferase	oxidative stress-related
*Sod*	superoxide dismutase	oxidative stress-related
*Frt*	ferritin	iron-metabolizing
*pepck*	phosphoenolpyruvate carboxykinase	gluconeogenesis
*ppar*	peroxisome proliferator-activated receptor	regulation of lipid metabolism
*pparg*	peroxisome proliferator-activated receptor γ	regulation of lipid metabolism
*tgfβ1*	transforming growth factor beta 1	cytokine
*tgfβr2*	transforming growth factor receptor type 2	cytokine
*Tnf*	tumor necrosis factor	cytokine
*tp53*	tumor protein p53	tumor associated
*myc*	transcription family	proto-oncogenes
*bc2*	B cell CLL/lymphoma 2	tumor associated
*tp53*	tumor protein 53 antigen	tumor associated
*erbB2*	HER2 family	tumor associated
*pcna*	proliferating cell nuclear antigen	cell proliferation
*ctnnβ*	protein associated with β-catenin	cell-cell adhesion, transcription
*gr*	glucocorticoid receptor	stress-related
*hsp70*	heat shock protein 70	stress-related
*fasl*	transmembrane protein	cell death, immunoregulation
*ifit1*	interferon induced protein	antiviral cytokine
*ifn1*	interferon alpha	antiviral cytokine
*il8*	interleukin-8	chemokine
*cxcl13*	C-X-C motif chemokine ligand 13	chemokine
*wshbv*	white sucker hepatitis B virus	pre-genomic RNA
*wshbvp a*	white sucker hepatitis B protein a	polymerase ORF
*wshbvp b*	white sucker hepatitis B protein b	polymerase ORF
*wshbvs*	white sucker hepatitis B virus s	S ORF polymerase
*ef1a*	elongation factor 1α	housekeeping gene
*etif3d*	eukaryotic translation initiation factor 3D	housekeeping gene
*rpl8*	ribosomal protein L8	housekeeping gene

### RNAscope methods

RNAscope (Advanced Cell Diagnostics, Newark, California) methods were developed to visualize the location (i.e., target cells) of the viral wshbvs gene (locus_tag = wsHBV_gp3, NCBI Gene ID: 26039967) of the WSHBV. Tissues were processed for histopathology, embedded into paraffin, and sectioned at 5 μm. Selected liver sections were deparaffinized with three changes of Pro-Par clearant (Anatech Ltd., Michigan) for 5 min each and rehydrated with a graded ethanol series of 100%, 95%, 80%, 70%, and 50% for 3 min each and air dried. RNAscope was conducted according to the manufacturer’s protocols for the RNAscope 2.5 HD Assay – RED (Wang et al. 2012). Next, the wsHBV_gp3 target probe was prepared by warming for 10 min at 40 °C, cooled to room temperature, and hybridization was carried out at 40 °C for 2 h in an InSlide Out hybridization oven (Boekel, Pennsylvania). The negative control slides consisted of the addition of probe diluent only. One dot per every 10 cells displaying background staining at 20× magnification was considered acceptable according to the manufacturer. All slides were counterstained with hematoxylin, rinsed clear with deionized water, and mounted with ProLongTM Gold Antifade Mountant (Thermo Fisher Scientific, Carlsbad, California).

### Statistical analyses

Data were compared using GraphPad Prism version 10.1.2 (GraphPad Software Inc., La Jolla, California). The Mann-Whitney test was used for comparisons of length, weight and age between sexes within a site and between sites. Data collected in 2021 was compared to that collected in 2012 (Blazer et al. 2017) and 2017 (Blazer et al. 2019) using the Kruskal-Wallis multiple comparison test. Prevalence of visible lesions and microscopically verified skin and liver neoplasia was compared between the sexes and sites using the Fisher exact test. A Spearman’s rank correlation analysis was used to test associations between viral transcripts and other hepatic transcripts. Hepatic transcript abundance was also analyzed for differences between groups (sex and site) using ROSALIND, a software designed for differential transcript expression analysis. A Generalized Linear Model (GLM) for count data, developed by the NanoString Biostatistics team, was employed by ROSALIND, which assumes a negative binomial distribution. Raw data, along with noise and dispersion estimates, were utilized by this model to calculate fold changes and adjusted p-values for each gene. The Fast method, as described in the nSolver Advanced Analysis 2.0 User Manual (https://nanostring.com/wp-content/uploads/MAN-10030-03_nCounter_Advanced_Analysis_2.0_User_Manual.pdf), was used for analysis, and to correct for multiple testing, the Benjamini-Hochberg false discovery rate (FDR) method was applied. Using this method, fold changes were derived from the statistical model rather than through direct mean comparisons between sample groups, incorporating noise and dispersion metrics across the dataset. Transcripts were considered significantly upregulated or downregulated with a fold change ≥ 1.5 or ≤ 1.5 and a false discovery rate (adjusted p-value) of ≤ 0.05. A *p-*value of 0.05 was used to indicate significance in all tests.

To account for demographic differences among fish samples (age, length, and sex) when evaluating tumor incidence, we used a Bayesian logistic regression framework based on Mahmood et al. (2014) and Rutter (2010). This approach allowed us to assess the relationship between tumor presence (skin and liver) and individual predictors while incorporating random effects for site and year where appropriate. Continuous predictors (age and total length) were standardized (mean = 0, SD = 1) to improve model convergence and comparability of effect sizes. Sex was included as a categorical variable. Length was used instead of weight as the morphometric predictor due to high correlation between the two and negligible differences in model estimates during preliminary runs.

Bayesian generalized linear models with a Bernoulli distribution and logit link were used to estimate tumor probability. Separate models were fit for each tumor type and each site (Sheboygan and Green Bay AOCs), in addition to a combined model that included random intercepts for site and year. To more accurately assess a potential decrease in tumor prevalence over time, the Bayesian logistic regression model was re-fit with year as a fixed effect for the Sheboygan AOC, using 2012 as the reference year, and adjusted for fish length, sex, and age. This allowed us to estimate the direct contrast between 2012 and 2021, rather than comparing each year to the overall mean. We employed two robust and complementary methods to evaluate this contrast: (1) Posterior draws from the model and (2) A formal hypothesis test using hypothesis() function.

The general structure of the combined model was: tumor presence ~ age + length + sex + (1 | site) + (1 | year). Posterior distributions were summarized using means and 95% credible intervals. Posterior probabilities, or probabilities of direction (PD), were summarized to represent the proportion of the posterior distribution that shares the same direction as the mean estimate. Models were implemented in R (v4.4.3) using the brms package and run with four chains of 2000 iterations each (1000 warm-up). Convergence was assessed via R̂ (≤ 1.01), effective sample size, and trace plots. Leave-One-Out cross-validation (via the loo package) was used to assess predictive performance. Key R packages included brms, rstan, tidyverse, bayesplot, MCMCvis, sjPlot, loo, cowplot, patchwork, and supporting packages for data manipulation and visualization.

## Results

### Morphometric and visible abnormalities

#### Green Bay AOC

**Fig. 3:**
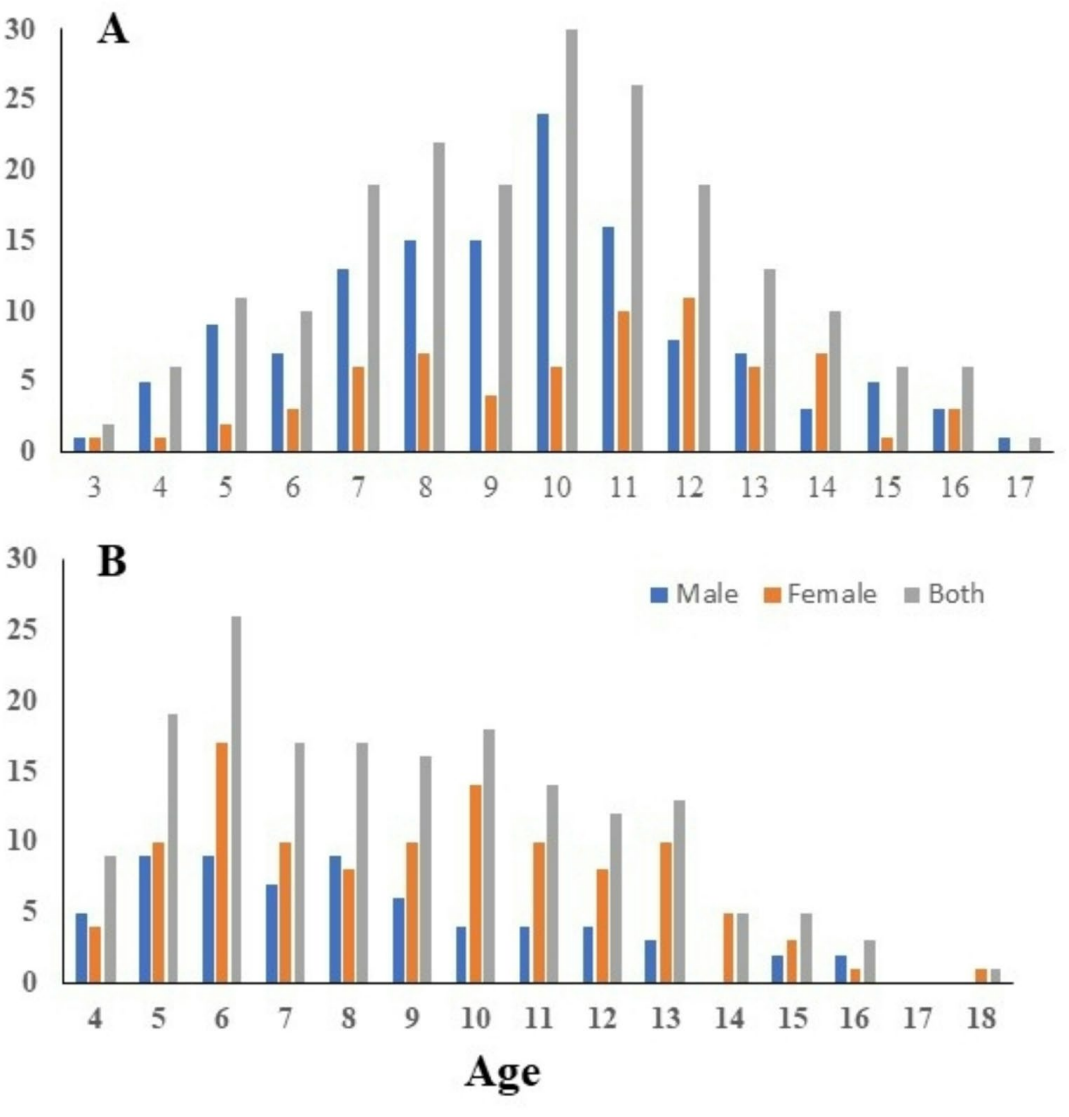
Age distribution or percentage of female, male and combined sexes at each age in years of white sucker *Catostomus commersonii* collected at the Green Bay (**A**) and Sheboygan (**B**) Areas of Concern in 2021

**Fig. 4:**
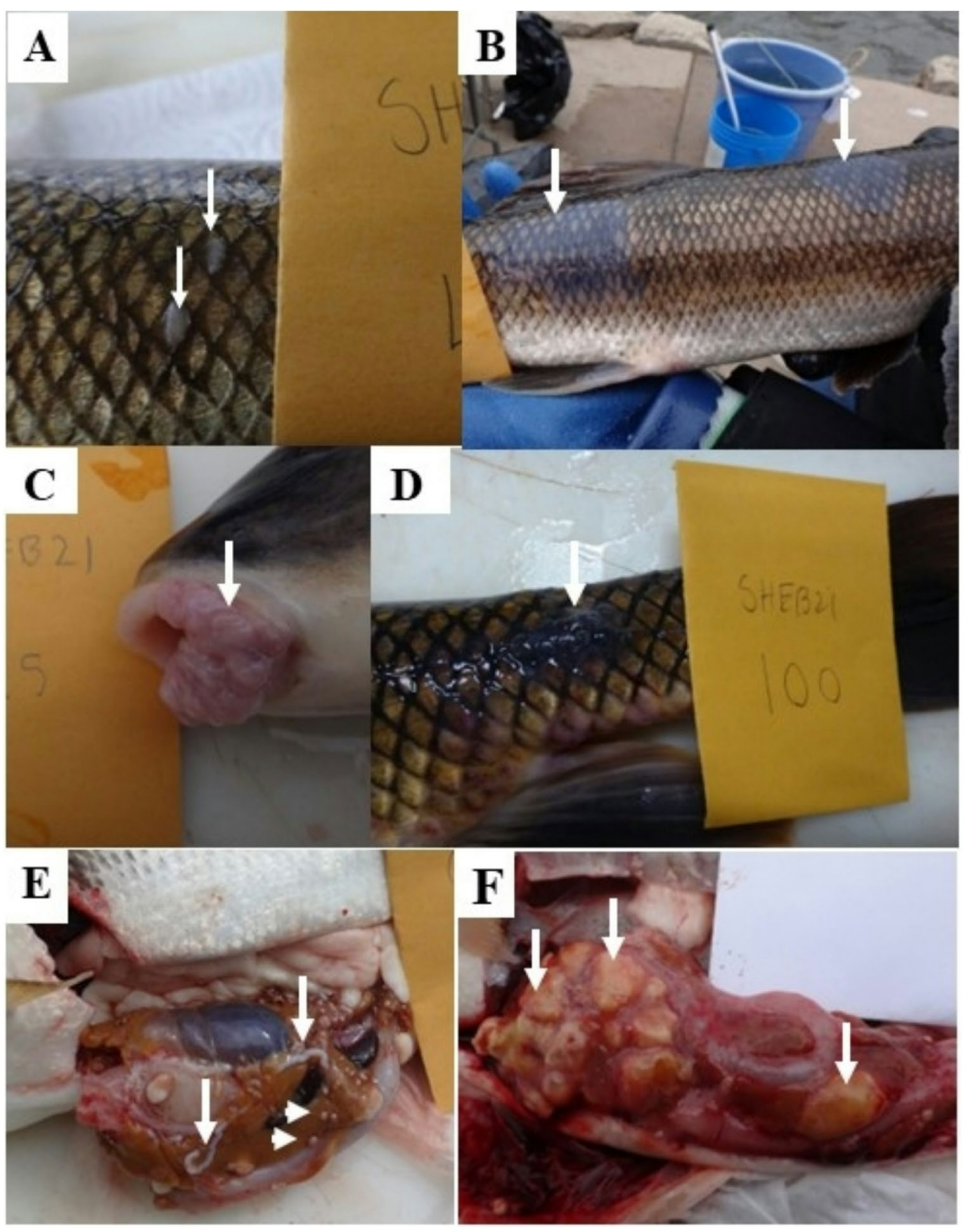
Visible abnormalities noted on white sucker *Catostomus commersonii* collected at the Sheboygan River and Green Bay Areas of Concern, April 2021. (**A**) Raised discrete white spots, often on an individual scale (arrows). (**B**) Slightly raised, diffuse, grayish, mucoid lesions on the body surface (arrows). (**C**) Raised lip lesions (arrow). (**D**) Raised body surface lesions (arrow). (**E**) White ribbon-like structures (arrows) and white cysts (arrowheads) on the liver and within the mesentery. (**F**) Nodules (arrows) throughout the liver tissue

At the Green Bay AOC 68 female and 132 male white sucker were collected. Age was determined for all fish and ranged from 3 to 17 years (Fig. 3A). Green Bay females were significantly (*p* = 0.0104) older than males. Females were also longer (*p* < 0.0001) and heavier (*p* < 0.0001) than males (Table 2). External abnormalities included discrete, small white spots (Fig. 4A), slightly raised mucoid (Fig. 4B) and raised, papillomatosis lip (Fig. 4C) and body (Fig. 4D) lesions. Additionally, raised, hard fin nodules were documented on 6.8% of the males and 11.8% of the females. Thin, red worms were observed in the fins of 12% of the fish. Small discrete white spots were observed in 2.0%, mucoid lesions in 11.0% and raised lip or body surface in 18% of the white sucker sampled. Raised skin lesions were only observed in females age 7 and above and in males age 5 and above. Although female suckers had a higher prevalence of raised lesions than males, and males had a higher prevalence of discrete white spots and mucoid lesions, there were no significant differences between the sexes (Table 2). White cysts of varying sizes and ribbon-like worms, identified as tapeworms, were also observed on the liver and within the abdominal cavity (Fig. 4E). No female and two males (1.5%) had visible liver nodules (Fig. 4F).

**Table 2:** Morphometric data (mean ± standard error) and percentage of white sucker *Catostomus commersonii* with external visible abnormalities collected at the Green Bay and Sheboygan river Areas of Concern (AOC) in 2021. Values within each AOC followed by a different superscipt indicate significant difference between sexes. Values for all fish followed by a different superscript indicate a difference between the sites. Number in parentheses is sample size

Site	Length (millimeter)	Weight (gram)	Age (year)	Discrete White (%)	Mucoid (%)	Raised (%)
**Green Bay**						
Female (68)	480.1 ± 3.6^a^	1237.0 ± 30.2^a^	10.4 ± 0.4^a^	0.0^a^	8.8^a^	23.5^a^
Male (132)	436.6 ± 1.7^b^	866.7 ± 10.9^b^	9.4 ± 0.3^b^	3.0^a^	12.1^a^	15.2^a^
All (200)	451.4 ± 2.2^A^	992.5 ± 17.6^A^	9.8 ± 0.2^A^	2.0^A^	11.0^A^	18.0^A^
**Sheboygan**						
Female (130)	478.0 ± 4.2^a^	1108.0 ± 23.7^a^	9.1 ± 0.3^a^	6.2^a^	7.7^a^	23.8^a^
Male (70)	427.8 ± 5.3^b^	792.1 ± 23.7^b^	8.2 ± 0.4^a^	2.9^a^	5.7^a^	22.9^a^
All (200)	460.4 ± 3.7^A^	997.7 ± 20.5^A^	8.8 ± 0.2^B^	5.0^A^	7.0^A^	23.5^A^

#### Sheboygan river AOC

At the Sheboygan AOC, 130 females and 70 males were collected and ranged in age from 3 to 18 (Fig. 3B). We were not able to determine age for 24 fish due to no or broken otoliths. There was no significant difference in age between female and male suckers, however females were longer (*p* < 0.0001) and heavier (*p* < 0.0001) than males (Table 2). Raised skin lesions were observed in males and females 5 years and older. Discrete white spots were observed in 5.0%, mucoid lesions in 7.0% and raised lesions in 23.5% of the white sucker. Female white sucker had a higher prevalence (but not significantly higher) of all three lesion types compared to male (Table 2). Red worms were observed in the fins of 8.5% of fish sampled. Two (1.5%) females and no males had visible liver nodules. Ribbon-like structures identified as tapeworms, adhesions and small white cysts were also observed on the liver and within the abdominal cavity.

The only significant difference between the sites when both sexes were combined was age. Green Bay white suckers were significantly older than those from the Sheboygan River (Table 2).

### Microscopic observations

The small white spots and mucoid lesions were primarily hyperplastic epithelial and potentially pre-neoplastic. A continuum from hyperplasia to benign neoplasia (papilloma) was observed. The larger, raised lesions were primarily papilloma, although squamous cell carcinomas were also observed (Table 3).

**Table 3:** Prevalence of preneoplastic and neoplastic microscopic lesions in white sucker *Catostomus commersonii* collected at the Green Bay and Sheboygan river Areas of Concern (AOCs) in 2021

	Papilloma	Carcinoma	Total Skin Neoplasms	Altered Foci	Hepatic Cell Neoplasms	Bile Duct Proliferation	Bile Duct Neoplasms	Total Liver Neoplasms
*Green Bay AOC - All*	*13.0*	*2.5*	*15.5*	*8.0*	*0.5*	*36.5*	*6.5*	*7.0*
Female	13.2	4.4	17.6	8.8	0.0	35.3	5.9	5.9
Male	12.9	1.5	14.4	7.6	0.8	37.1	7.6	7.6
*Sheboygan River AOC - All*	*18.0*	*3.5*	*21.5*	*4.0*	*2.0*	*54.5*	*4.0*	*6.0*
Female	19.2	3.1	22.3	3.8	3.1	53.8	3.8	6.9
Male	15.7	4.2	20.0	4.2	0	55.7	4.3	4.3

**Fig. 5:**
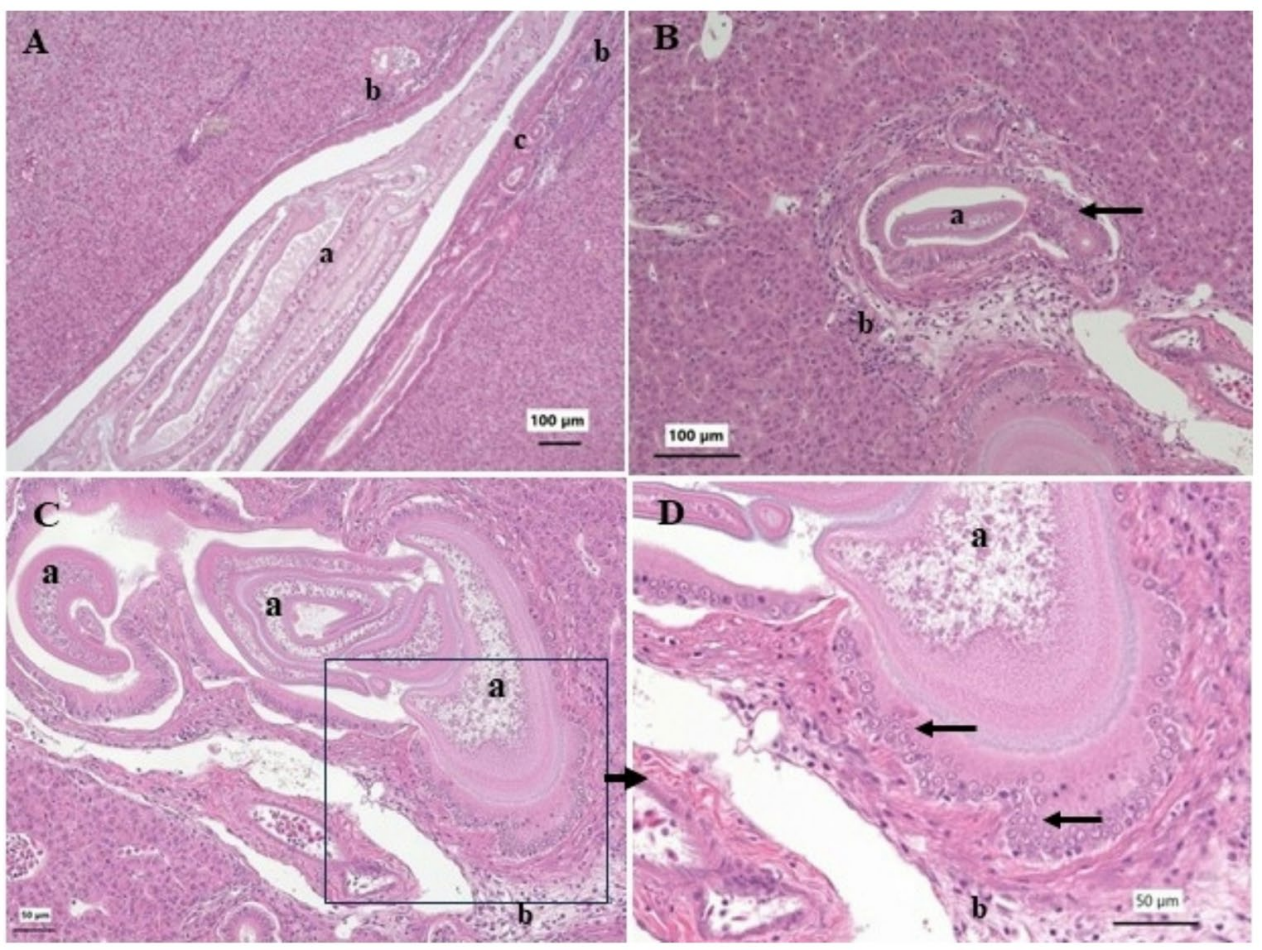
Microscopic lesions of bile ducts in white sucker *Catostomus commersonii* A. Longitudinal section of a bile duct containing myxozoan plasmodia (a). Inflammation (b) and bile duct proliferation (c) are evident around the affected bile duct. B. Transverse cross section of a bile duct containing a myxozoan plasmodium (a). Inflammation and fibrosis (b) are evident. C. Multiple myxozoan plasmodia (a) within distended bile ducts. Surrounding tissue contains inflammation and fibrosis (b). Box indicates area illustrated in D. Higher magnification of the bile duct containing myxozoan plasmodia (a), proliferation of bile duct epithelial cells with thickening of the bile duct epithelium (arrows) and inflammation and fibrosis in the surrounding tissue. Hematoxylin and eosin stain

**Fig. 6:**
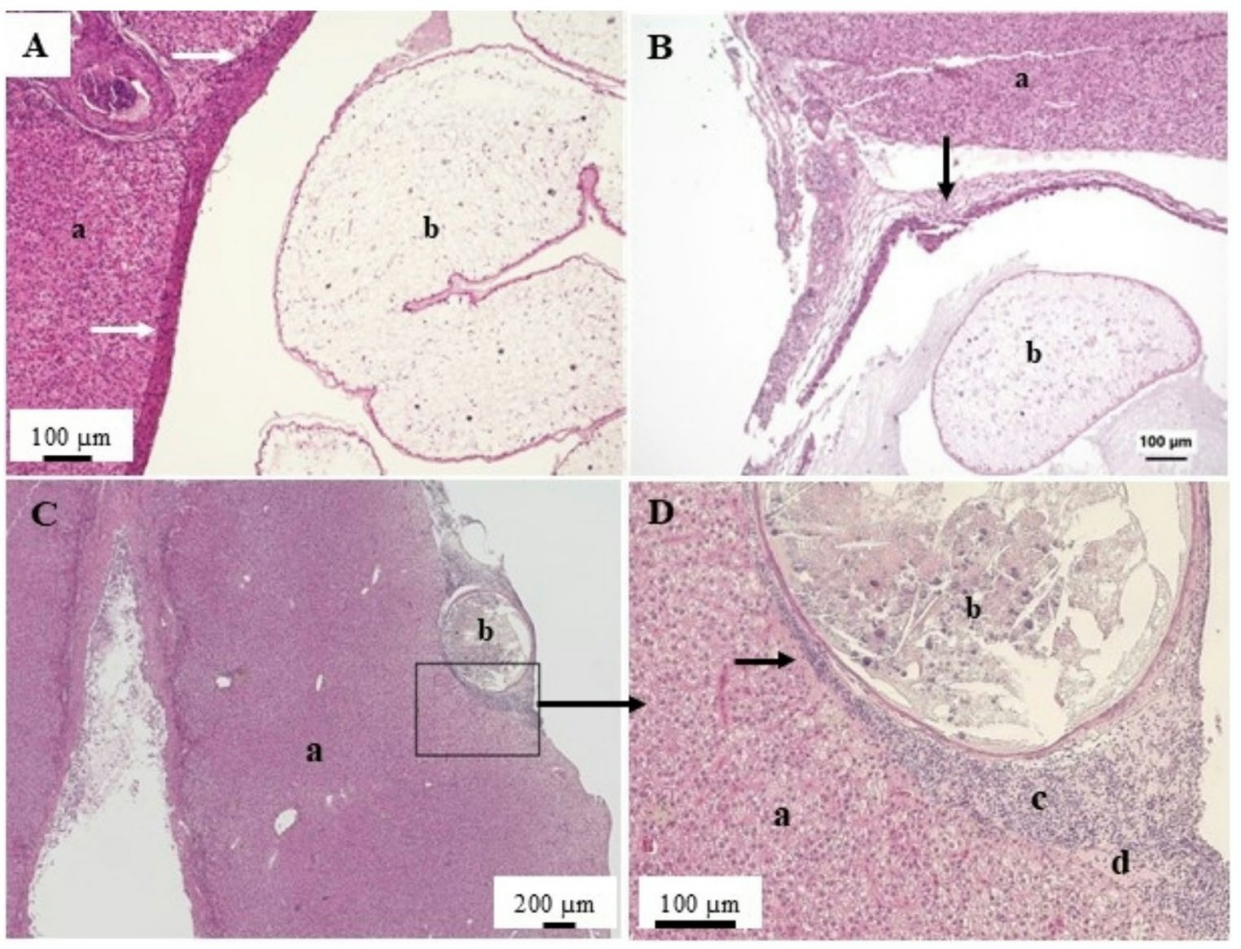
Microscopic liver changes associated with parasites. A. Cyst within the liver (a) containing an unidentified cestode pleurocercoid (b). Encysted parasite causes compression of hepatocytes and inflammation (arrows). B. Encysted pleurocercoid (b) causing inflammation and fibrosis (arrow) within liver (a). C. Section of liver (a) containing a cyst with necrotic tissue (b). D. Higher magnification of area within the box illustrating liver tissue (a) with cyst (b) and associated inflammation (c), macrophage aggregate (d) and hepatocyte necrosis (arrow). Hematoxylin and eosin stain

Liver lesions documented included bile duct proliferation, foci of cellular alteration and both hepatic (hepatic cell adenoma, hepatic cell carcinoma) and bile duct (cholangioma, cholangiocarcinoma) neoplasms. Bile duct proliferation was the most common hepatic microscopic observation (Table 3) and ranged from mild to severe. In some liver sections the bile duct proliferation coincided with the observation of plasmodia of a myxozoan parasite. In longitudinal sections of the bile ducts the plasmodia could inhabit a considerable length (Fig. 5A). Bile duct proliferation and inflammation were observed along the affected bile duct. More commonly, cross sections of the bile ducts were observed (Fig. 5B and C). The presence of plasmodia was associated with inflammation, bile duct proliferation (Fig. 5A-C) and proliferation of the bile duct epithelial cells leading to a thickening of the epithelium (Fig. 5D). In some areas signs of dysplasia were observed, including epithelium stratification, cellular pleomorphism and increased cellular density (Fig. 5D). Large cysts containing cestode pleurocercoids were observed on the surface and within liver tissue. Compression of the surrounding hepatocytes and inflammation were observed in response to the encapsulated parasites (Fig. 6A and B). Smaller cysts containing necrotic tissue were also observed. The cause of these was not determined. Often inflammation, edema and fibrosis were associated with these cysts (Figs. 6C and D).

### Prevalence of microscopic lesions

#### Green Bay AOC

Skin neoplasms (papilloma or squamous cell carcinoma) were observed in males age 5 and older, but only in females 8 and older. The prevalence of verified skin neoplasms was 15.5%, 2.5% were squamous cell carcinoma and 13.0% were papilloma. Liver neoplasms were observed in male white sucker 8 years and older and females 9 years and older. Overall prevalence of liver neoplasms was 7% and all but one were of bile duct origin (Table 3). One male had both a cholangiocarcinoma and hepatic adenoma.

#### Sheboygan River AOC

Neoplastic skin lesions were documented in suckers 6 years of age and older. The prevalence of neoplastic skin lesions was 21.5%, 3.5% squamous cell carcinoma and 18.0% papilloma. Liver neoplasms were observed in females ages 9 and above and males ages 10 and above. Overall prevalence was 6%, 2% of hepatic cell and 4% of bile duct origin. Male white sucker from this site did not have any neoplasms of hepatic cell origin (Table 3).

### Temporal changes in neoplasms at the Sheboygan AOC

In 2012, 193 suckers were collected and in 2017 and 2021, 200 were collected. In both 2012 and 2021 more females than males were collected while in 2017, there were more males. While the mean age of suckers collected in 2012 was older than the other two collection years, mean lengths were not significantly different (Table 4), perhaps suggesting better growth in later years.

**Table 4:** Temporal comparison of age and length, presented as mean ± standard error, of white sucker *Catostomus commersonii* from Sheboygan River Area of Concern, 2012–2021. Values within each group followed by the same superscipt are not significantly different

	Sample Size	Collection Year	Age (year)	Total Length (millimeter)
All Fish	193	2012	10.2 ± 0.3^a^	458.8 ± 4.6^a^
	200	2017	8.6 ± 0.2^b^	457.9 ± 2.6^a^
	200	2021	8.8 ± 0.2^b^	460.4 ± 3.7^a^
Female	112	2012	10.4 ± 0.4^a^	475.8 ± 6.4^a^
	77	2017	8.4 ± 0.3^b^	474.8 ± 4.8^a^
	130	2021	9.1 ± 0.3^ab^	478.0 ± 4.2^a^
Male	81	2012	10.0 ± 0.5^a^	435.4 ± 5.5^ab^
	120	2017	8.7 ± 0.3^ab^	447.3 ± 2.5^a^
	70	2021	8.2 ± 0.4^b^	427.8 ± 5.3^b^

The prevalence of skin neoplasms decreased significantly (*p* = 0.0168) from a high of 32.6% in 2012 to a low in 2021 of 21.5%. Papilloma prevalence decreased with each sampling period, while squamous cell carcinomas were highest in 2017. Liver neoplasms were 8.3 and 8.5% in 2012 and 2017, respectively and decreased, although not significantly, to 6.0% in 2021. Bile duct neoplasms and hepatic cell adenoma prevalence decreased over the sampling periods while hepatocellular carcinoma increased (Table 5).

**Table 5:** Temporal comparison of the percentage of white sucker *Catostomus commersonii* with neoplastic lesions at Sheboygan River Area of Concern

Neoplastic Lesions	2012	2017	2021
*Total Skin Neoplasms*	*32.6*	*29.5*	*21.5*
Papilloma	30.5	20.0	18.0
Squamous cell carcinoma	2.1	9.5	3.5
*Total Liver Neoplasms*	*8.3*	*8.5*	*6.0*
Cholangioma	2.6	1.0	0.8
Cholangiocarcinoma	3.6	3.5	2.3
Hepatic cell adenoma	1.0	2.0	0.5
Hepatocellular carcinoma	1.0	2.0	2.3

### Modeling of sample demographics and tumor incidence

Bayesian logistic regression models identified fish length as the most consistent and influential predictor of neoplasia presence across both liver and skin tissues. In the combined model (data collected from both AOCs), longer fish had significantly higher odds of neoplasia, with 95% credible intervals that did not overlap one (Supplemental Table 2; Supplemental Fig. 1A). Male fish also showed increased odds of skin neoplasia, while the effect of sex on liver neoplasia was weaker and less certain. Age was moderately associated with liver neoplasia but had no meaningful relationship with skin tumors.

Effect estimates were generally consistent across site-specific models (Sheboygan AOC and Green Bay AOC), though the strength and certainty of associations varied (Supplemental Fig. 1B–C). Length remained a strong predictor in most models, while the effects of sex and age were more variable between sites. Random effects for site and year were centered near zero with wide, overlapping credible intervals, indicating no substantial residual differences in neoplasia risk between sites or across sampling years, after accounting for individual fish characteristics (Supplemental Fig. 2A–B). However, point estimates for year-level effects in the skin neoplasia overall model (SHEB + GB) suggested a potential decrease in skin tumor occurrence over the study period of the Sheboygan AOC. To investigate this, the model was re-fit specifically for the Sheboygan AOC to determine the odds of a decrease. Year was included as a fixed effect, using 2012 as the reference year, and adjusting for fish length, sex, and age. Two complementary approaches to evaluate the contrast between 2012 and 2021 were used; posterior draws from the model and a formal hypothesis test. Both approaches provided strong and consistent evidence for a significant decline in skin neoplasia from 2012 to 2021 at the Sheboygan AOC. The estimated odds ratio was 0.58, corresponding to a 42% reduction in odds, with 90% and 95% credible intervals ([0.38, 0.88] and [0.35, 0.96], respectively), and posterior probabilities of a decrease exceeding 98%. In contrast, liver neoplasia showed a decreasing trend with an estimated 36% reduction in odds (odds ratio = 0.64), but the 95% credible interval (0.24 to 1.59) included 1. Posterior probabilities (83%) and evidence rations (4.81) suggest insufficient evidence to conclude a statistically significant decrease, consistent with the overall model’s point estimates (Supplementary Fig. 2B).

### Liver transcript abundance

The transcript abundance of 36 genes, plus three housekeeping genes, were measured in liver tissue of white sucker from each site (Table 1). The mean abundance was compared for male and female white sucker at each site (Supplemental Tables 3 and 4). No reference site or control data was available for comparison, but transcript abundance was high for genes associated with oxidative stress (*gst*, *sod*), contaminant exposure (*cyp1a1*, *mt2*), estrogenic endocrine regulation (*era*,* erb2*), iron-metabolism and the immune response (*frt*) and *pepck* a gene associated with glucose metabolism (Table 6).

**Table 6:** Hepatic transcript abundance in white sucker *Catostomus commersonii* liver tissue collected from the Sheboygan River and Green Bay Areas of Concern in 2021. Data are presented as mean ± standard error and values within each area of concern followed by different superscipts indicate a significant difference between sexes

Transcript	Female Abundance	Male Abundance	Female Abundance	Male Abundance
	*Sheboygan Area of Concern*		*Green Bay Area of Concern*	
*erα*	6,496 ± 239^a^	5,763 ± 239^b^	18,960 ± 866^a^	12,081 ± 479^b^
*erβ2*	6,326 ± 237^a^	6,555 ± 314^a^	45,797 ± 2,785^a^	28,205 ± 1,158^b^
*cyp1a1*	60,998 ± 3,232^a^	87,620 ± 4,081^b^	39,505 ± 3,871^a^	93,780 ± 5,065^b^
*mt2*	68,831 ± 3,192^a^	116,554 ± 6,876^b^	111,908 ± 5,185^a^	109,255 ± 4,000^a^
*gst*	96,314 ± 5,088^a^	137,906 ± 5,975^b^	48,082 ± 2,109^a^	73,242 ± 2,441^b^
*sod*	11,417 ± 397^a^	24,343 ± 1,306^b^	13,403 ± 644^a^	18,193 ± 537^b^
*frt*	175,373 ± 5,172^a^	288,349 ± 9,495^b^	289,413 ± 10,540^a^	429,420 ± 8,412^b^
*pepck*	6,300 ± 303^a^	9,796 ± 395^b^	32,293 ± 1,994^a^	39,759 ± 1,245^b^
*wshbv*	1,694 ± 946^a^	478 ± 189^b^	341 ± 57^a^	607 ± 245^b^
*wshbv a*	105,041 ± 23,901	36,116 ± 16,700	31,139 ± 21,638^a^	45,961 ± 24,602^a^
*wshbv b*	67,768 ± 15,647	17,604 ± 7,197	21,533 ± 14,697^a^	41,146 ± 20,837^a^
*wshbvs*	82,851 ± 19,296	20,801 ± 8,716	20,885 ± 13,964^a^	51,381 ± 25,808^b^

**Fig. 7:**
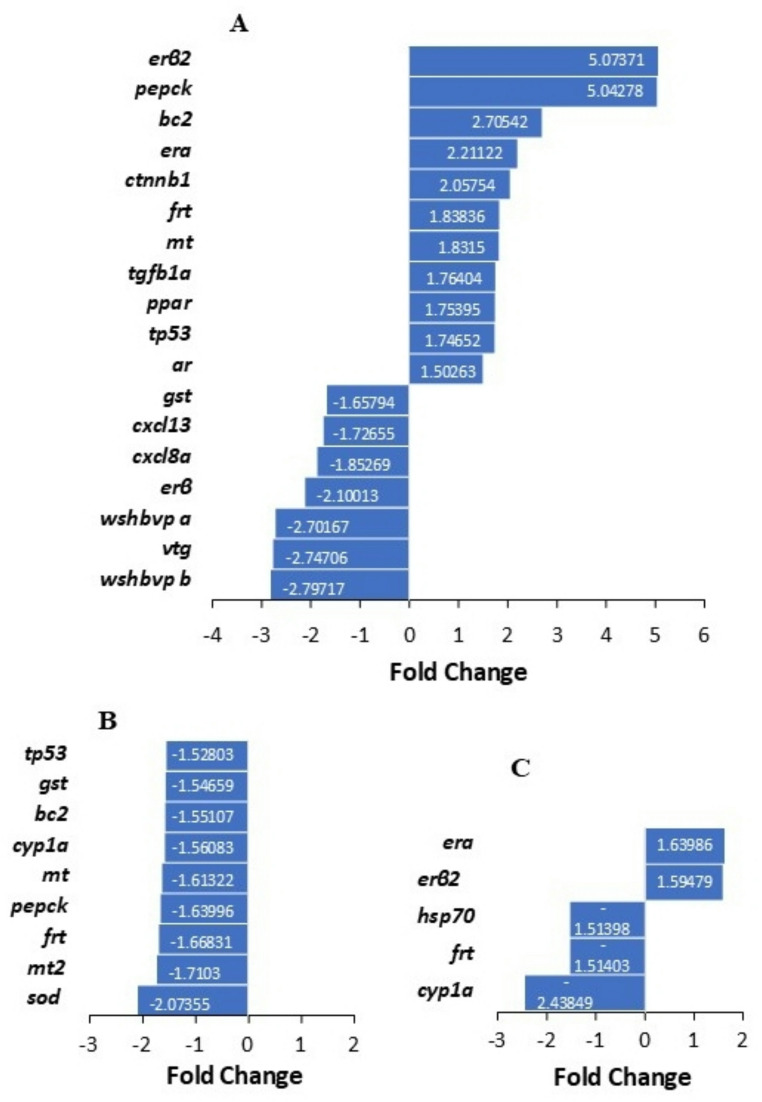
Differential expression in liver transcript abundance of white sucker *Catostomus commersonii* for (**A**) Green Bay Area of Concern (AOC) versus the Sheboygan River AOC; (**B**) females versus males at the Sheboygan River AOC, and (**C**) females versus males at the Green Bay AOC

Utilizing fold-change comparisons, eleven genes were upregulated at the Green Bay AOC compared to the Sheboygan River AOC (Fig. 7A), including *mt*, *erα*,* erβ2*,* ar*, *pepck*, *frt*, *tgfb1a* (associated with the immune response), *bc2*,* tp53* (cancer pathways), transcription (*ctnnb1*, *ppar*), while seven genes were downregulated (Fig. 7A), including genes associated with the white sucker hepatitis virus (*wshbvp b*,* wshbvp a*), estrogenic endocrine disruption (*erβ*,* vtg*), oxidative stress (*gst*), and the immune response (*cxcl8a*,* cxcl13*).

Genes related to contaminant exposure (*cyp1a1*) and iron metabolism (*frt*) were down-regulated in females at both sites (Fig. 7B-C). At the Sheboygan River AOC, seven more genes were downregulated in females compared to males (Fig. 7B), including more genes associated with contaminant exposure (*mt*,* mt2*) plus genes associated with cancer pathways (*bc2*,* tp53*), oxidative stress (*gst*,* sod*), and glucose metabolism (*pepck*). At the Green Bay AOC, stress-related *hsp70* was also down-regulated in females compared to males and estrogen receptors (*erα*,* erβ2*) were upregulated (Fig. 7C).

Four sequences of the WSHBV were included in the codeset. The *wshbv* transcript was lower than the other three transcripts, with only 23% of the samples above 200 transcripts (level of detection was 50 transcripts). Conversely, 15% of *wshbvp a*, *wshbvp b* and *wshbvs* polymerase gene transcripts were greater than 22,000 with counts of *wshbvp a* reaching 1,364,092, *wshbvp b* reaching 729,956 and *wshbvs* reaching 971,422. These three transcripts were significantly correlated which is expected. At the Sheboygan site viral transcript abundance was higher in females than males, however at the Green Bay site males had higher mean abundance for all transcripts but only *wshbv* was significantly higher (Table 6).

Several liver transcripts, combining males and females, were correlated with the presence of the WSHBV, utilizing the *wshbvp a* transcript (Table 7). The abundance of 10 transcripts were significantly correlated at both sites. These included transcripts related to immunity (*ifn1*, *tnf*, *fasL*), tumor or cell proliferation (*tp53*, *pcna*), stress and contaminant exposure (*ahr*, *hsp70*) and transcription (*ctnnb1*). At the Sheboygan AOC an additional five liver transcripts were correlated with the viral transcript and included three immune-related (*ifit1*, *tgfbr2*, *cxcl13*) and two stress or contaminant-related (*mt2*, *gr*). Three additional liver transcripts were correlated with the viral transcript at Green Bay AOC including *mt*, *ar*, and *pepck* (Table 7).

**Table 7:** Significant correlations of the transcript abundance of *Wshbvp a* with other hepatic transcript sequences in white sucker *Catostomus commersonii* collected from the Sheboygan and Green Bay Areas of Concern (AOCs) in 2021

Transcript	Sheboygan River AOC		Green Bay AOC	
	Spearman ρ	p-value	Spearman ρ	p-value
*ifn1*	0.4175	< 0.0001	0.2634	0.0002
*hsp70*	0.4026	< 0.0001	0.2524	0.0004
*ahr*	0.3848	< 0.0001	0.2623	0.0002
*ppar*	0.3808	< 0.0001	0.2344	0.0009
*tnf*	0.3807	< 0.0001	0.0169	0.0179
*fasL*	0.3563	< 0.0001	0.2136	0.0027
*tp53*	0.3333	< 0.0001	0.2245	0.0016
*pcna*	0.2463	0.0006	0.1952	0.0061
*ctnnβ1*	0.1760	0.0146	0.1522	0.0332
*frt*	0.1495	0.0385	0.2234	0.0016
*ifit1*	0.3676	< 0.0001		ns
*tgfbr2*	0.3105	< 0.0001		ns
*gr*	0.2846	< 0.0001		ns
*cxcl13*	0.2868	< 0.0001		ns
*mt2*	0.2415	0.0007		ns
*mt*		ns	0.3021	< 0.0001
*ar*		ns	0.2965	< 0.0001
*pepck*		ns	0.2340	0.0010

### RNAscope results

**Fig. 8:**
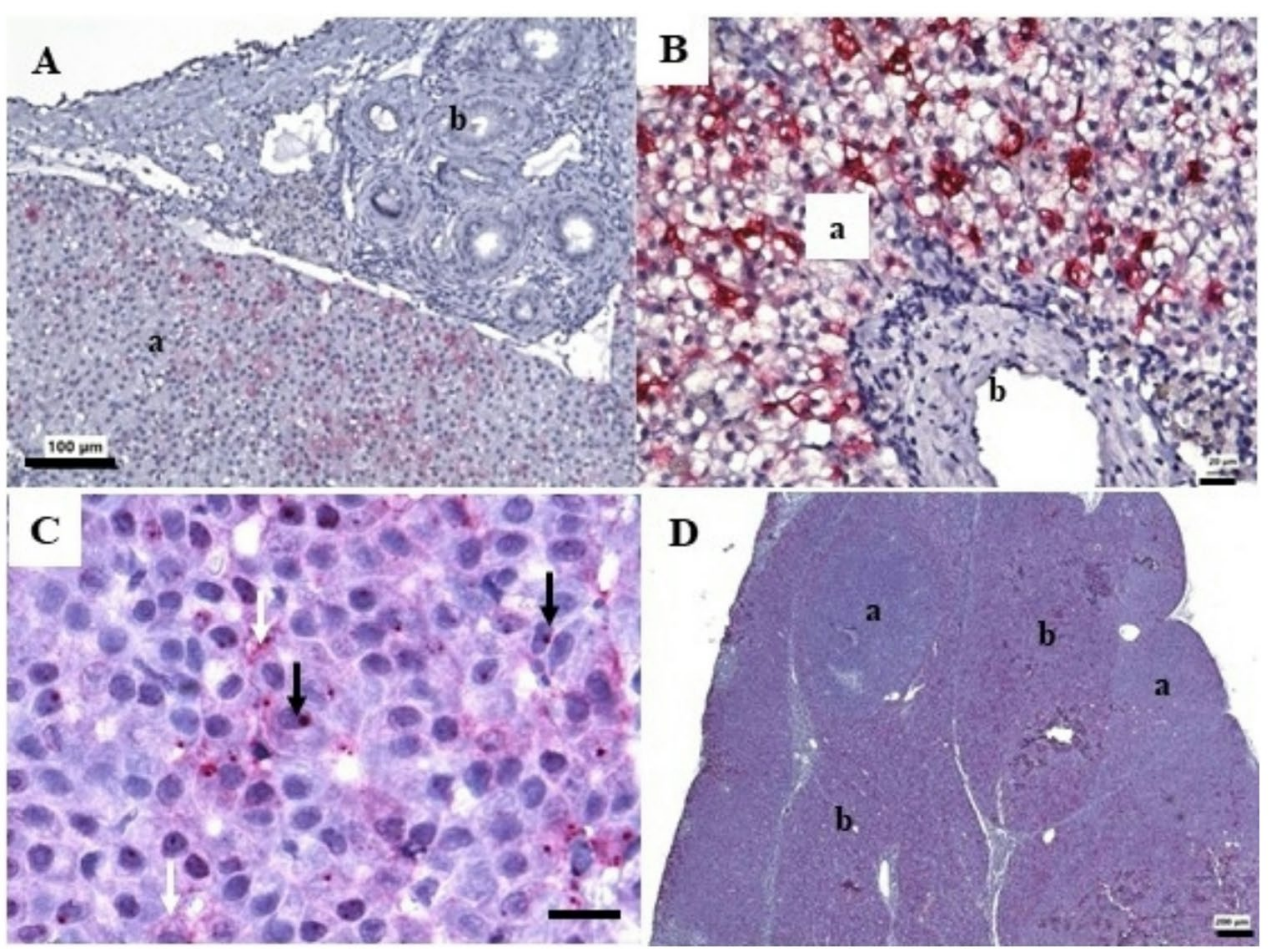
Microscopic results of RNAscope in situ hybridization of microscopic lesions in liver of white sucker *Catostomus commersonii*. **A**. Hepatic tissue (a) contains positive-staining cells while areas of bile duct proliferation (b) are negative. Scale bar equals 100 μm. **B**. Higher magnification of infected hepatocytes (a) and uninfected bile duct (b). Scale bar equals 20 μm. **C**. Positive staining was noted in the nuclei of some hepatocytes (black arrows) and the cytoplasm of others (white arrows). Scale bar equals 20 μm. **D**. Nodules of hepatocellular carcinoma (a) within normal hepatic tissue (b). Increased positive staining intensity within normal tissue (b). Scale bar equals 200 μm. Fast red positive staining illustrates viral presence. Hematoxylin and eosin counter stain

RNAscope in situ hybridization was used to localize viral RNA (*wshvps*). Positive staining revealed the presence of viral RNA in hepatocytes but was absent in normal bile ducts or areas of bile duct proliferation (Fig. 8A and B). In liver tissue with a high abundance of viral transcripts, hepatocytes with positively-staining material, primarily in the cytoplasm, were evident (Fig. 8B). In other sections, positive staining was evident in the nuclei and cytoplasm (Fig. 8C). Hepatitis B viruses reside within cell nuclei and replicates in cytoplasm (Beck and Nassal 2007), hence these observations may be due to the replication cycle and activity of the virus in specific samples. In a section of hepatocellular carcinoma (Fig. 8D) positive staining was primarily located in areas of normal hepatic tissue.

## Discussion

Skin and liver tumors have been used as indicators of environmental health in numerous fish species worldwide (Brown et al. 1973; Malins et al. 1987; Vogelbein et al. 1990; Pinkney et al. 2009; Stentiford et al. 2005; Lerebours et al. 2014). White sucker, an indicator species for the fish tumor BUI in the Great Lakes watershed, is a demersal species and hence may be exposed to contaminants that accumulate in sediments. In riverine studies white sucker have shown limited mobility most of the year but move up- or downstream to smaller tributaries during spawning (Doherty 2004; Doherty et al. 2010). To our knowledge spatial and temporal movements of white sucker in the Sheboygan and Fox Rivers have not been studied. However, in the Boardman River, another Lake Michigan tributary, adult white sucker migrated into the river in the spring for spawning and embryo incubation (Swanson et al. 2021). They return and aggregate in near-shore lake areas with slow to moderate velocity from summer to late winter (Doherty et al. 2010). In our studies mature adults were collected during the spring spawning migration and it is not known exactly where they spend the remainder of the year.

Historically skin and liver neoplasms at Great Lakes AOCs have been associated with chemical contaminants (Baumann 1992; Baumann et al. 1996; Blazer et al. 2009a, b a, b; Rafferty et al. 2009; Baines et al. 2021). An early summary of studies on skin and liver neoplasms in white sucker and brown bullhead *Ameiurus nebulosus* from sites throughout the Great Lakes suggested that skin neoplasm prevalence above 25% and hepatic neoplasm prevalence above 5% is indicative of environmental degradation (Baumann et al. 1996). More recently some researchers have suggested skin neoplasms should not be used as indicators of chemical carcinogenesis since viruses had been noted in these lesions. While there has been some evidence of a viral etiology, to our knowledge no specific virus has been definitively associated with these neoplasms in white sucker, particularly the squamous cell carcinomas. Papillomas in white sucker were reported to proliferate in crowded, laboratory conditions, but regress in non-crowded conditions (Premdas and Metcalfe 1994). Laboratory studies have also shown that injections of testosterone and 17β-estradiol induced papillomas, suggesting the initiation and development of these neoplasms are multifactorial (Premdas et al. 2001). Our analysis showed a significant decrease in skin tumors between 2012 and 2021 at the Sheboygan AOC. Further studies focused on identification of potential viruses and environmental factors that may influence mucosal immunity could help to understand the development of papilloma and squamous cell carcinoma in this species.

The “fish tumors and other deformities” is one of the remaining BUIs at the Green Bay and Sheboygan River AOCs. Previous monitoring at the Green Bay AOC involved a variety of species. Brown et al. (1973) collected 2121 fish of multiple species and documented a liver neoplasia incidence of 4.4% when including all species. However, in brown bullhead the incidence was 12.2%. A later study utilized walleye (*Sander vitreus*) collected in the Fox River and four sites within Green Bay during 1996–1997. A liver neoplasia prevalence of 8.4% in 83 adults ranging in age from 4 to 11 years was observed, while in walleye age 5–8, there were no liver neoplasms in females (*n* = 23), but 17% of males (*n* = 41) had neoplasms. No liver neoplasms were observed in the reference areas (Barron et al. 2000). In the current study one male white sucker had both a hepatic cell and bile duct neoplasm and no females had a hepatic cell neoplasm, resulting in overall occurrence of 0.5%. The majority of tumors observed were bile duct neoplasms and males had a slightly higher occurrence than females (Table 3). In walleye collected in 1996–1997 only hepatocellular neoplasms and altered foci were reported and were related to PCB concentrations (Barron et al. 2000). It is not known if the difference in results (hepatic versus bile duct) in the two studies is a consequence of species differences, changes in contaminant exposure or other risk factors such as parasites.

Sediment dredging and remediation in the Green Bay AOC was completed in 2020 and involved PCB cleanup and removal of sediment with PAHs and heavy metals (Lower Green Bay and Fox River Area of Concern Beneficial Use Impairment Removal Recommendation: Restrictions on Dredging, 2021; https://widnr.widen.net/view/pdf/yylcjunver/GW_LGB_ DredgingBUIRemoval2021.pdf? t.download = true). We observed liver neoplasms in fish eight years of age and older, hence in fish spawned before the last dredging and present during dredging activity. At the Sheboygan River AOC liver neoplasm prevalence was 8.3% in 2012, 8.5% in 2017 and down to 6.0% in 2021. Projects to remediate contaminated sediments and restore habitat in the Sheboygan River AOC were completed in 2013 and included dredging within areas of the river and harbor to remove contaminated sediments (htpps://www.epa.gov/ great-lakes-aocs/remediation-and-restoration-projects-sheboygan-river-aoc). Consequently, the majority of white sucker collected in 2017 were spawned prior to or during sediment cleanup, as were those ages 8 and older in 2021 (age groups with liver neoplasms). Hence, at both sites, the white sucker age classes observed with tumors could have been exposed during the latest dredging activities. A previous study with brown bullhead in the Black River, Ohio reported the prevalence of liver neoplasms to be 22 to 39% while a coking plant was operating. Tumor prevalence fell to 10% after closure of the plant and associated declines of PAH concentrations in sediment, however 2–4 years after dredging (in 1992–1993) the prevalence was greater than 45% (Baumann 1998).

No white suckers were collected from reference or control sites in the current study. However, in 2012, 200 white suckers were collected at the Kewaunee River, a non-AOC Lake Michigan tributary. The occurrence of liver neoplasia at this site was 3.5%, with 2.5% bile duct tumors and 1.0% hepatic cell tumors (Blazer et al. 2017). Overall, liver neoplasia occurrence was higher (7.0%) at Green Bay and Sheboygan (6.0%) in the current study. There were higher bile duct neoplasms (6.5%) at Green Bay with most in male suckers and higher hepatic cell neoplasms at Sheboygan (2.0%) compared to Green Bay (0.5%) with female suckers having a higher prevalence. These results may suggest different risk factors associated with initiation or progression at the two sites, differences between hepatic and bile duct carcinogenesis and between the sexes. However, there were also differences between the sites in age structure and sex ratio of fish sampled. Logistical modeling combining both sites did indicate, as previously observed that age was associated with liver neoplasia. Males showed increased odds of skin neoplasia, but sex had less of an effect on liver neoplasia.

Previous studies have varied in findings of sex differences in both responses to environmental contaminants and tumors prevalence. Baumann et al. (1987) did not find a sex difference in the prevalence of liver neoplasms, mostly of bile duct origin, in brown bullhead collected from a contaminated site in the Great Lakes drainage. Conversely, a higher prevalence of liver neoplasia was observed in female brown bullhead in Chesapeake Bay tributaries (Pinkney et al. 2019). No difference was noted between the sexes of neoplasms in English sole (*Parophrys vetulus*) used to monitor effects in Puget Sound (Rhodes et al. 1987). In flounder (*Platichthys flesus*) used for monitoring health effects of contaminants in the North and Baltic Seas, females had a higher frequency of hepatocellular origin (Koehler 2004).

We utilized a set of genes previously developed (Hahn et al. 2016) to identify contaminant, endocrine and immune-related responses that may assist in identifying risk factors for liver carcinogenesis in white sucker, as well as explain sex and site differences. Gene expression indicates a recent response to environmental factors and physiological responses, while carcinogenesis often involves initiation of cellular changes followed by promotion to neoplastic changes. Exposure to initiators and promoters may be separated by long time periods, hence gene expression at the time of capture will not provide a definitive explanation of “cause” but may help to identify risk factors.

While legacy contaminants have certainly been associated with neoplasia in fish, particularly liver tumors, there is an increasing recognition of the importance of considering pathogens and parasites, alone or in conjunction with exposure to chemical carcinogens and/or chemicals that cause immunosuppression, in carcinogenesis (Dheilly et al. 2019; Hatta et al. 2021). Both legacy contaminants such as PCBs, PAHs and metals, as well as emerging contaminants (Sahoo et al. 2021) may be associated with neoplastic changes. The high abundance of certain gene transcripts at both sites suggests there may be ongoing exposure to legacy contaminants, as well as endocrine disrupting chemicals. Up-regulation of *cyp1a1*, which has been used as a biomarker of exposure to PAHs, co-planar PCBs and other contaminants including xenoestrogens (Williams et al. 1998; Bucheli and Fent 1995; Kopecka-Pilarczyk and Schirmer 2016) was observed. High transcript abundance of metallothionein (*mt2*) and indicators of oxidative stress such as *gst* and *sod*, used in many studies as a markers of heavy metal exposure (Espinoza et al. 2012; Kim and Kang 2016) were also observed. *Mt* is a protein important in heavy metal detoxification, scavenges free radicals to mitigate oxidative damage and hence can have an anticancer effect and reduce inflammation (Yang et al. 2024). Ferritin (*frt*) is protein involved in iron metabolism and storage is upregulated in oxidative stress and inflammation (Neves et al. 2009; Zheng et al. 2010). In addition to genes associated with exposure to legacy contaminants, male white sucker had high abundance of estrogen receptor transcripts (*era*, *erb2*). In male bass species (*Micropterus spp.*) collected at Great Lakes AOCs, abundance of both these transcripts was correlated with the presence of testicular oocytes, a biomarker for exposure to estrogenic endocrine disruptors (Blazer et al. 2018). A study within the Green Bay AOC included chemical analyses and effects-based monitoring using caged fathead minnow (*Pimephales promelas*) at multiple sites. Estrogenic activity was detected at most sites and *cyp1a1* expression was increased in minnow liver tissue (Li et al. 2017). In addition to reproductive effects, estrogenic endocrine disruptors are known to influence the immune response of fishes (Milla et al. 2011; Sinha and Mandal 2025). Like Green Bay, liver transcripts related to contaminant exposures (*cyp1a1*, *mt2*, *gst*,* sod*) were abundant at the Sheboygan site. Estrogen responsive gene transcripts in males (*era*, *erb2*) were also increased although not as high as males at Green Bay (Table 6).

Sex differences in expression of genes related to contaminant exposure and oxidative stress could partially explain differences in neoplasm prevalence. Male white sucker had a higher abundance of *cyp1a1*, *gst* and *sod* at both sites, while abundance of *mt2* was only significantly higher in males from the Sheboygan River site. Gene expression in response to heavy metal exposure was found to be sex-specific in wild Nile tilapia (*Oreochromis niloticus*). Expression of *gst* and *mt* were higher in liver of tilapia males at all three locations monitored (Awad et al. 2024).

Infectious agents can contribute to carcinogenesis in a number of ways, including inflammation and cell damage in chronic infections, induction of oncogenic gene expression and disruption of immunological recognition (Dalton-Griffin and Kellam 2009; Hatta et al. 2021). Risk factors for neoplasms in other fishes include viruses and parasites (Anders and Yoshimiza 1994; Coffee et al. 2013; Vergneau-Grosset et al. 2017; Matsche et al. 2020). Hence, in this study we assessed biological factors possibly associated with liver neoplasia. Tapeworms or cestodes are common parasitic worms that as adults are endoparasites in the digestive tract but larvae (pleurocercoids or metacestodes) can occur in the body cavity or within various organs. Migrating metacestodes cause damage including inflammation, fibrosis, necrosis and edema (Scholz et al. 2021). The cestode observed in our study was not identified although a number of species have been identified from white sucker. Larval *Triaenophorus nodulosus* have been found in white sucker from the Great Lakes (Muzzall and Whelan 2011). The inflammation, fibrosis and necrosis associated with these organisms could contribute to carcinogenesis.

Two liver trematode parasites (*Opisthorchis viverrine*, *Clonorchis sinensis*) are major risk factor for bile duct tumors in humans (Sripa et al. 2012; Suk et al. 2018), contributing to carcinogenesis via mechanical injury to biliary epithelial cells, inflammation, production of cytokines and chemokines and release of oxygen free radicals that eventually cause DNA damage and tumorigenesis (reviewed by Hatta et al. 2021). While we did not observe trematodes in the bile ducts of white sucker we did observe a bile duct myxozoan associated with inflammation, as well as proliferation of bile duct epithelial cells. Recently, a bile duct myxozoan together with chemical contaminant exposure was associated with biliary tumors in white perch (*Morone americana*) from Chesapeake Bay tributaries (Matsche et al. 2020).

Another possible risk factor for liver neoplasia is the WSHBV, a member of the genus *Parahepadnavirus*, within the Hepadnaviridae. Hepatitis B viruses (HBV) are small, enveloped DNA viruses that reside within cell nuclei and replicate in the cytoplasm (Beck and Nassal 2007). Until relatively recently they had only been reported from mammals and birds (Dill et al. 2016). Chronic infections of the liver with HBV in humans and other homeotherms is associated with an increased risk of hepatocellular carcinoma. Virulence is affected by host specific factors (age of exposure, route of exposure, immune status) and viral genotypes (Kramvis and Kew 2007; Chauhan and Michalak 2021). Phylogenetic analysis of the WSHBV genome from 27 virus positive white sucker collected in United States tributaries of Lakes Michigan, Superior and Erie and the Athabasca River in Canada indicated phylogeographic diversity and five haplotypes were identified (Adams et al. 2021). The WSHBV lacks the X protein (Hahn et al. 2015) usually associated with induction of neoplasia (Kew 2011). In other animals HBV infection has been shown to induce intracellular production of reactive oxygen species and oxidative stress (Popa and Popa 2022). Two transcripts *sod* and *gst* associated with oxidative stress were abundant in white sucker liver tissue, although were not correlated with transcript abundance of WSHBV. There was also no correlation between viral loads and preneoplastic or neoplastic lesions even when a subset of age and sex matched individuals without microscopic lesions were compared to those with various lesions. In situ hybridization demonstrated the presence of viral RNA within hepatocytes but not within in bile duct epithelial cells, so WSHBV is unlikely a co-factor in the biliary neoplasms observed. The role of WSHBV in proliferative hepatic cell lesions, alone or in conjunction with contaminants is currently unknown. In mammalian hosts studies indicate hepatocarcinogenesis is a long-term, multistage process that involves multiple rick factors (Chen and Chen 2002), which may also be the case for white sucker.

A number of liver transcripts were significantly correlated with transcript abundance of WSHBV at both sites, indicating a response to the virus. This included interferon 1 (*ifn1*) an innate antiviral cytokine (Gan et al. 2020) and *hsp70*, a heat shock protein which plays a role in the immune response and inflammation in fish (Roberts et al. 2010). Heat shock proteins are important in numerous mammalian viral diseases including HBV (Lubkowska et al. 2021) and *hsp70* actively facilitates HBV capsid assembly (Seo et al. 2018). Our results suggest it may be an important factor in the WSHBV infections as well. The interactions between contaminant exposure and cellular damage due to parasite and viral pathogens is currently unknown.

In conclusion, hepatic neoplasm occurrence was 6 and 7% respectively at the Sheboygan River and Green Bay AOCs. A reference or control site for the monitoring in 2021 was not included, however, both are above the 3.5% documented at a non-AOC site in the Lake Michigan watershed in 2012 (Blazer et al. 2017). Hepatic transcript abundance of genes associated with chemical exposure indicate ongoing exposure to legacy and emerging contaminants that may influence immune responses and carcinogenesis. Additionally, the detection of WSHBV transcripts, inflammatory markers, and parasite-associated lesions highlights the potential for complex interactions between chemical and biological factors in development of liver neoplasms. Identifying potential viral and environmental factors associated with the development of skin neoplasms in white sucker also remains a research gap. The odds of a skin tumor significantly decreased between 2012 and 2021 at the Sheboygan site, perhaps suggesting different risk factors than those for liver tumors. Further monitoring and assessment focusing on individuals spawned after the completion of sediment remediation and including similar age white sucker collected at a reference site could help determine the effectiveness of restoration efforts and reveal potential emerging impacts to be addressed.

## Supplementary Information

Below is the link to the electronic supplementary material.

Supplementary Information

## Data Availability

Data are available in Blazer et al. (2025) at https://doi.org/10.5066/P1SHMAZP.
