# Supplementary material for: Assessment of the fish tumors or other deformities beneficial use impairment and associated risks at two Lake Michigan Areas of Concern
Source: Ecotoxicology. 2026 Jan 3;35(2):31. doi: 10.1007/s10646-025-03001-8 (PMC13233900; doi:10.1007/s10646-025-03001-8)
Supplement: Supplementary file 1 — Supplementary Information [file 10646_2025_3001_MOESM1_ESM.docx]

**Supplemental Table 1.** Gene sequences included in the white sucker *Catostomus commersonii* liver codeSet.

| **Gene Symbol** | **Target sequence** |
| --- | --- |
| *ahr* | AGTACTTTCAATGGGATGTAACCACTGGCACCATGTGTACAGAATAATTTTTGAAGGAAGTCTAGAGCTTCAGAGGTTCACAGATGTTCTGGATGAATAT |
| *ar* | CCGTGCAGACATGGGGTTGCATCGGGGCGCCGAATCAGGATGGAAATGCAGCGGTGCCGCTGAAGTTCTGTCACGCGAGTTGGGTTTGAGTCGTGACGCA |
| *cat* | TCACACGTTATTGCAAAGCCAAACTGTTTGAGCATGTGGGAAAAAGGACACCCATCGCTGTTCGCTTTTCCACTGTGGCTGGGGAGTCTGGGTCAGCAGA |
| *ctnnβ1* | AGTATCCAGTGGAGGGACTGCCTGACTTGGGTCACGCTCAGGACCTGATGGACGGCTTGCCACCCACAGACTCAAATCAGCTGGCCTGGTTCGACACAGA |
| *ef1a* | AGTCAACCACCACTGGTCATCTGATCTACAGATACTGGACAAAGGGGAGCAGCAGCCGAGGAGTGATCTCTCAACACTGAAACCAATCAATCATGGGGAA |
| *eif3d* | CGGAGGTGGAAGCTTGGGGAAGATATTGACCTCATTGTTCGCTGCGAGCATGATGGAGTGATGACTGGAGCAAACGGAGAAGTGTCTTTCATCAACGTGA |
| *erα* | CTTTATGCATGCCTTCAGAGCATGTGCTGGTGTTACTTTTAGGTGCTGAGCCACCAGCTCTCTGCTCGCGACAGAAACAAAACCGCCCATACACCGAGAT |
| *erβ* | CAATGGAAACGAGGTTGTTACCGTGGCGTTAAGCCCAGAGGAACTAATAGCTCGCATCATAGATGCAGAGCCACCACAGATTTACCTTATGAAAGATATG |
| *frt* | GAAACTGATGAAGTTTCAGAACCAGAGGGGAGGGAAGATCTTTCTGCAGGATGTGAAAAAACCAGAGAAGGATGAGTGGGGAAGTGGTTTGGAAGCTCTT |
| *gr* | AAATGCTCGCAGAGATCATCAGCAACCAGTTACCAAAATTCAAAGCTGGGAGTGTCAAACCGCTACTCTTTCATCAGAAGTGAAGCTCCTCCCTGCAGCA |
| *gst* | AGGATGGTGACCTGGTCCTGTATCAGTCCAATACTATGTTAAGACATTTGGGTCGCAAACATGGTGCATATGGGAAAAATGACTCTGAGGCTTCTCTTAT |
| *hsp70* | GACGATTGGAAATGCTGGGAAGAGCCAGATAATAACTAATTTTAAGAACACAGTTCACGGCTTTAAGAAGTTGCATGGTCGCACATTCGATGACCCTTAC |
| *mt* | TACACGAACAGTTAGTGCACTTGCAGGTGGCACCACAGTTGCAAGTTCCAGTCTTGGAGCAATCACAAGGATCCATTTTCCCTCAAAATATCCTTAAAGG |
| *pcna* | AAGATGCCTTCTGGTGAATTTGCCCGAATCTGCAGAGACCTGTCACAGATTGGTGACGCTGTCATGATTTCCTGTGCCAAAGATGGAGTGAAGTTCTCTG |
| *pepck* | GTTGGGGCCCGGTCCCTCTCCTCCATCCCCTCTTTGCCTCCATCTGTGGCTGAGTTTGTGTCAGGGGCCGCATCTGAGTGTAAACCTGCCAAAGTGCACT |
| *ppar* | CTTGATGACAGCGACCTTGCTCTGTTTGTGGCAACTGTCATACTGTGTGGAGATCGTCCTGGACTGATGAACGTGAAACAGGTAGAGCAGATCCAGGATG |
| *rpl8* | CACAACCCTGAGACCAAAAAGTCCAGAGTTAAGCTTCCATCTGGATCCAAAAAGGTTGTCTCTTCCTCAAACAGAGCTGTAGTTGGTGTCGTTGCTGGTG |
| *sod* | CAAAATTCAGAGAGGCAGCATGGAACCAAAAAGGGCTGTTTGTGTGCTCAAAGGCACAGGGGAAGTGACCGGAACAGTCTATTTCGACCAAGCGGATGAC |
| *tgfβ1* | GTTTGATGTGAAGAAGTCAATCACAGAATGGCTGCAAAGTTCAGAAGATGAAGTGAGTTTAGATTTGAGGTTATACTGTGGCTGTAAAACCGGCAAAGAC |
| *tgfβr2* | ATTTGCATAGTGACCACACACCCTGTGGGAGGCCCAAAGTGCCCATCGTCCACCGAGATCTAAAGAGTTCCAACATACTGGTGAAGAATGATCTTTCCTG |
| *tnf* | GATTGCAGTCCTTTTCAGGCACAATCGGTTCAGTGATGAGGAGCTAAAGTTGGCTCAGACCTTCAAAAAGAAACTGCACCAGGGAGCCATGACAGCCATC |
| *tp53* | TGAGGAAGAGAATCTAAAGAAGATTACTGGGGAGAAGTCTACTGGGACAAAGAGAAAACGTCAGCTTCAGGATCAGTTGAAAGAGCATCCTATACCTGAT |
| *vtg* | TATGCTGGTGACCGTTCCTCTGAGAACACTGATCCTGAAAGGCCTGCTGTGAGAACTGCTACTCCATTCCACAAGACCCTCTGTCTTGATGTCCCATACA |
| *bc2* | CAATGGGAGATGAAGATGATGAAGAGGAGAGTGATGCTGTTGTATCTCAAGTTCTTGATGAGCTGGGTCTGACACTTTCTGATGAACTTTCAAACCTGCC |
| *cxcl13* | AGCGCACATATTTACTGCTGGCAGCTGTTACAATCTGCTGCTTCACAAATCTGCTTGCTGTCTCAATGGAAGGCTTTACATCCAATAACAAGTGTCACTG |
| *cyp1a1* | AATATTCTTGTGCTCTTGAGGAGCACGTCATCAAAGAGGGACTCTATCTGATAGAAAGATTACACACTGTCATGAAGGCTGATGGGAGCTTCGATCCTTT |
| *ef1a* | GCTGTGCTGATTGTTGCTGGTGGTGTTGGTGAGTTCGAGGCTGGTATCTCCAAGAACGGACAGACCCGTGAGCATGCCCTTCTGGCTTTTACATTGGGAG |
| *erb2* | CCTGCTGATGCTGCTGTCACATATACGACACCTCAGCAACAAAGGTATTGAGCATCTGTCGAGCATGAAGAGAAAAAACGTGGTGCTTCTGTACGATCTT |
| *erbB2* | CCCGGTCAGTGTGTGTCATGTGTCAATTACAAACGTGGGACCGAGTGTGTGGAGCAGTGTAATGTCCTACATGGGTCAGTACGGGAGTTTGTGGATGGTT |
| *fasl* | ATGGATACTGGCTTTGATACTCCTGTTGGTTTTTGCAGCCCTGGGACTGGGAGCCTACCAGATACTGAGGTTGCAGACTGAATTTGAAAGGCTGACACAG |
| *ifit1* | ACATGTGATTAGATATGTGGGGAAATATATGCGGGTCTACGGGTCTTTAGATAGGTCCACTGCCCTGTTGAAAAGAGCATTGGAGGTTGCACCAAATTCA |
| *ifn1* | AAGGACTATGATTGAGCAACGGGTGATGGTCTTTTTCTGGGGAGTATGTTTGTTGACTTCTGGATGGATGACATACGCCGATGCCAGCATCCCTGAAAAC |
| *il8* | GATGAAGTTCACTGTAGCAGCTTTCACCATTCTGATCTGCATGACGCTACTGTCCACAACAGAAAGTAGATCCCTACAACAACTACGTTGCAACTGTATT |
| *mt2* | ACCTGCAAGTGCACTAATTGTGCGTGTACAACCTGCAAGAAGAGTTGCTGCTCCTGCTGCCCATCCGGTTGCAGCAAGTGTGCTTCTGGCTGTGTATGTA |
| *myc* | GATGATAGCTGACAATACAGATAGCAGCTCGAATCGACACAATGCAAGTTATTTGCAGGATCTGAGCACTTCTGCATCAGAATGCATCGATCCTTCGGTG |
| *pparg* | CTACTCCGAAGAGAGCATCTACAGAGCACAAGAATCCCAAAATTCAATCAAACTTGAACCCGAGTCTCCTCCACAGTTTGCAGATAACAGCCTGTCATTT |
| *tp53* | AGAAAAAGTGAGGAGAGCAACTTTAGAAAAGAGCAGGAGGCCAAAACCATGGGAAAGACCTCCTCTGCCACCAAACGAAGCTTTAAGGAGTCATCCTCAT |
| *wshbv* | CCGTCTTAAAGCCGTGTAAGCACGGAGAGCGAAAAGCAAAGGAAGAGTTTTGAATTGTTTGTAGCAAACAAACATAGAGTCAGATGCGAGTGCGCGACAG |
| *wshbvp a* | AACAGTTACGCTCCACAAAGAATTGCCTCTTTTACTGCCTTCAAGGACTTGGAAATACTTGCTGCCGGCCAGGTTTTGGCCAAAGGTGACCCATTGGTAT |
| *wshbvp b* | AGATCTTCCTCACGACTTTTTCCCTCTGCTTAAAGATCAGGTACAGTTCTCTAAGGACGTTATCAAGGAGTATAACGAACACCATTCTCAGAATAGACAC |
| *wshbvs* | CAACCTCGATGCACTTCAAAACGGACTGCCCGCTAACATGTACCGGATTTCGCTGGACCTTAGTGAGGCGTTCTATCATATTCCTTTGCATCCTGGCTCT |

**Supplemental Table 2.** Posterior estimates of regression coefficients from Bayesian logistic regression models predicting liver and skin neoplasia presence in fish. Each row presents the posterior mean estimate (Value), 95% credible interval (Lower and Upper 95% CI), and the Probability of Direction (PD), which reflects the proportion of the posterior distribution that shares the same sign as the mean estimate. The intercept represents the expected log-odds of neoplasia when all predictors are zero; however, this is not always biologically meaningful for variables like length, age, and sex. Interpretation is more appropriately focused on the direction and magnitude of the predictor coefficients. Rows where the 95% CI does not include zero are bolded to identify significant effects.

| **Model** | **Tissue** | **Predictor** | **Value** | **Lower**  **95% CI** | **Upper**  **95% CI** | **PD** |
| --- | --- | --- | --- | --- | --- | --- |
| **Combined** | **Liver** | Intercept | -2.39 | -4.31 | 0.68 | 0.95 |
|  |  | Sex:Male | 0.15 | -0.62 | 0.94 | 0.65 |
|  |  | Age (years) | 0.30 | -0.03 | 0.63 | 0.96 |
|  |  | **Length (mm)** | **0.71** | **0.21** | **1.25** | **1.00** |
| **Combined** | **Skin** | Intercept | -1.48 | -3.39 | 0.97 | 0.93 |
|  |  | **Sex:Male** | **0.81** | **0.37** | **1.24** | **1.00** |
|  |  | Age (years) | 0.03 | -0.19 | 0.25 | 0.60 |
|  |  | **Length (mm)** | **0.62** | **0.35** | **0.93** | **1.00** |
| **Sheboygan** | **Liver** | Intercept | -1.92 | -5.39 | 2.58 | 0.85 |
|  |  | Sex:Male | 0.00 | -0.93 | 0.96 | 0.51 |
|  |  | Age (years) | 0.16 | -0.24 | 0.55 | 0.79 |
|  |  | **Length (mm)** | **1.07** | **0.40** | **1.75** | **1.00** |
| **Sheboygan** | **Skin** | Intercept | -1.16 | -3.97 | 2.19 | 0.83 |
|  |  | **Sex:Male** | **0.99** | **0.51** | **1.50** | **1.00** |
|  |  | Age (years) | -0.03 | -0.29 | 0.21 | 0.60 |
|  |  | **Length (mm)** | **0.78** | **0.45** | **1.15** | **1.00** |
| **Green Bay** | **Liver** | Intercept | -1.83 | -6.34 | 2.94 | 0.80 |
|  |  | Sex:Male | 0.47 | -1.12 | 2.21 | 0.70 |
|  |  | **Age (years)** | **0.67** | **0.05** | **1.27** | **0.98** |
|  |  | Length (mm) | -0.10 | -0.97 | 0.71 | 0.59 |
| **Green Bay** | **Skin** | Intercept | -0.93 | -5.51 | 3.20 | 0.69 |
|  |  | Sex:Male | -0.09 | -1.17 | 1.08 | 0.58 |
|  |  | Age (years) | 0.25 | -0.18 | 0.69 | 0.88 |
|  |  | Length (mm) | 0.04 | -0.53 | 0.61 | 0.55 |

**Supplemental Table 3.** Hepatic transcript abundance (mean ± standard error) in white sucker *Catostomus commersonii* collected from the Green Bay Area of Concern in 2021.

| Transcript | | Females | Males |
| --- | --- | --- | --- |
| Androgen receptor | *ar* | 1,999 ± 102 | 1,914 ± 61 |
| Estrogen receptor α | *erα* | 18,960 ± 866 | 12,081± 479 |
| Estrogen receptor β | *erβ* | 835 ± 53 | 1,167 ± 61 |
| Estrogen receptor β2 (Er-δ) | *erβ2* | 45,797 ± 2785 | 28,205 ± 1158 |
| Vitellogenin | *vtg* | 272,842 ± 18980 | 583 ± 174 |
| Aryl hydrocarbon receptor | *ahr* | 236 ± 10 | 233 ± 12 |
| Cytochrome P450 protein | *cyp1a1* | 39,505 ± 3871 | 93,780 ± 5065 |
| Metallothionein | *mt* | 129 ± 4 | 162 ± 3 |
| Metallothionein 2 | *mt2* | 111,908 ± 5185 | 109,255 ± 4000 |
| Glutathionine S-transferase | *gst* | 48,082 ± 2109 | 73,242 ± 2441 |
| Superoxide dismutase | *sod* | 13,403 ± 644 | 18,193 ± 537 |
| Ferritin | *frt* | 289,413 ± 10540 | 429,420 ± 8412 |
| Phosphoenolpyruvate carboxykinase | *pepck* | 32,293 ± 1994 | 39,759 ± 1245 |
| Peroxisome proliferator-activated receptor | *ppar* | 68 ± 5 | 80 ± 6 |
| Peroxisome proliferator-activated receptor γ | *pparg* | 2,840 ± 142 | 3,146 ± 103 |
| Transforming growth factor β1 | *tgfβ1* | 656 ± 20 | 799 ± 18 |
| Transforming growth factor receptor type 2 | *tgfβr2* | 571 ± 24 | 817 ± 29 |
| Tumor necrosis factor | *tnf* | 80 ± 4 | 116 ± 8 |
| Tumor protein p53 | *tp53* | 372 ± 10 | 460 ± 11 |
| Transcription family | *myc* | 328 ± 12 | 400 ± 15 |
| B cell CLL/lymphoma 2 | *bc2* | 9,544 ± 357 | 10,477 ± 252 |
| HER2 family | *erbB2* | 2,058 ± 92 | 2,016 ± 42 |
| Proliferating cell nuclear Ag | *pcna* | 417 ± 14 | 511 ± 25 |
| Protein associated with β-catenin | *ctnnβ* | 1,053 ± 37 | 1,358 ± 30 |
| Glucocorticoid receptor | *gr* | 1,224 ± 34 | 1,391 ± 25 |
| Heat shock protein 70 | *hsp70* | 27 ± 4 | 43 ± 5 |
| Transmembrane protein | *fasl* | 37 ± 4 | 57 ± 4 |
| Interferon induced protein | *ifit1* | 172 ± 16 | 223 ± 15 |
| Interferon alpha | *ifn1* | 32 ± 4 | 43 ± 5 |
| Interleukin-8 | *il8* | 1,509 ± 191 | 2,202 ± 234 |
| C-X-C motif chemokine ligand 13 | *cxcl13* | 212 ± 58 | 395 ± 64 |
| White sucker hepatitis B virus | *wshbv* | 341 ± 57 | 607 ± 245 |
| White sucker hepatitis B virus protein a | *wshbvp a* | 31,139 ± 21,638 | 45,961 ± 24,602 |
| White sucker hepatitis B virus protein b | *wshbvp b* | 21,533 ± 14,697 | 41,146 ± 20,837 |
| White sucker hepatitis B virus s | *wshbvs* | 20,885 ± 13,964 | 51,381 ± 25,808 |

**Supplemental Table 4.** Hepatic transcript abundance (mean ± standard error) in white sucker *Catostomus commersonii* liver tissue collected from the Sheboygan Area of Concern in 2021.

| Transcript | | Females | Males |
| --- | --- | --- | --- |
| Androgen receptor | *ar* | 1,280 ± 64 | 1,393 ± 56 |
| Estrogen receptor α | *erα* | 6,496 ± 239 | 5,763 ± 239 |
| Estrogen receptor β | *erβ* | 2,281 ± 130 | 2,184 ± 123 |
| Estrogen receptor β2 (Er-δ) | *erβ2* | 6.326 ± 237 | 6,555 ± 314 |
| Vitellogenin | *vtg* | 71,607 ± 8,822 | 2,450 ± 943 |
| Aryl hydrocarbon receptor | *ahr* | 235 ± 13 | 236 ± 16 |
| Cytochrome P450 protein | *cyp1a1* | 60,998 ± 3,232 | 87,620 ± 4,081 |
| Metallothionein | *mt* | 73 ± 2 | 116 ± 5 |
| Metallothionein 2 | *mt2* | 68,831 ± 3,192 | 116,554 ± 6,876 |
| Glutathionine S-transferase | *gst* | 96,314 ± 5,088 | 137,906 ± 5,975 |
| Superoxide dismutase | *sod* | 11,417 ± 397 | 24,343 ± 1,306 |
| Ferritin | *frt* | 175,373 ± 5,172 | 288,349 ± 9,495 |
| Phosphoenolpyruvate carboxykinase | *pepck* | 6,300 ± 303 | 9,796 ± 395 |
| Peroxisome proliferator-activated receptor | *ppar* | 47 ± 4 | 43 ± 2 |
| Peroxisome proliferator-activated receptor γ | *pparg* | 3,179 ± 102 | 3,701 ± 161 |
| Transforming growth factor β1 | *tgfβ1* | 392 ± 11 | 507 ± 17 |
| Transforming growth factor receptor type 2 | *tgfβr2* | 527 ± 25 | 684 ± 30 |
| Tumor necrosis factor | *tnf* | 96 ± 6 | 110 ± 7 |
| Tumor protein p53 | *tp53* | 236 ± 6 | 1,436 ± 77 |
| Transcription family | *myc* | 451 ± 23 | 536 ± 28 |
| B cell CLL/lymphoma 2 | *bc2* | 3,238 ± 89 | 5,116 ± 241 |
| HER2 family | *erbB2* | 1,378 ± 39 | 1,744 ± 58 |
| Proliferating cell nuclear antigen | *pcna* | 525 ± 37 | 635 ± 28 |
| Protein associated with β-catenin | *ctnnβ* | 574 ± 26 | 771 ± 39 |
| Glucocorticoid receptor | *gr* | 859 ± 21 | 1,149 ± 37 |
| Heat shock protein 70 | *hsp70* | 35 ± 3 | 35 ± 3 |
| Transmembrane protein | *fasl* | 52 ± 6 | 46 ± 4 |
| Interferon induced protein | *ifit1* | 158 ± 10 | 201 ± 16 |
| Interferon alpha | *ifn1* | 49 ± 6 | 41 ± 4 |
| Interleukin-8 | *il8* | 2,873 ± 170 | 3,493 ± 333 |
| C-X-C motif chemokine ligand 13 | *cxcl13* | 873 ± 136 | 876 ± 237 |
| White sucker hepatitis B virus | *wshbv* | 1,694 ± 946 | 478 ± 189 |
| White sucker hepatitis B virus protein a | *wshbvp a* | 105,041 ± 23,901 | 36,116 ± 16,700 |
| White sucker hepatitis B virus protein b | *wshbvp b* | 67,768 ± 15,647 | 17,604 ± 7,197 |
| White sucker hepatitis B virus protein vs | *wshbvs* | 82,851 ± 19,296 | 20,801 ± 8,716 |

**Supplemental Figure 1. Posterior mean odds ratios (OR) with 40% and 80% credible intervals for predictor variables from Bayesian logistic regression models of neoplasia presence in liver (left) and skin (right) for A) Combined model, B) Sheboygan AOC, and C) Green Bay AOC.** Predictors include standardized age (zage), length (zlength), and sex (sexM; male relative to female). In the liver neoplasia model, longer fish (OR = 2.10) show a strong positive association with neoplasia risk, while age (OR = 1.38) and male sex (OR = 1.26) have weaker, more uncertain effects. In the skin neoplasia model, both length (OR = 1.89) and male sex (OR = 2.30) are strongly associated with increased odds of neoplasia, whereas age (OR = 1.03) shows no meaningful effect. Credible intervals that do not overlap the vertical reference line at OR = 1 indicate stronger evidence for an association. The 40% and 80% Bayesian credible intervals represent ranges where the true parameter values likely fall, given the data and model. The numeric value is the Bayesian Point Estimate (BPE) mean, or the average value of the posterior distribution.


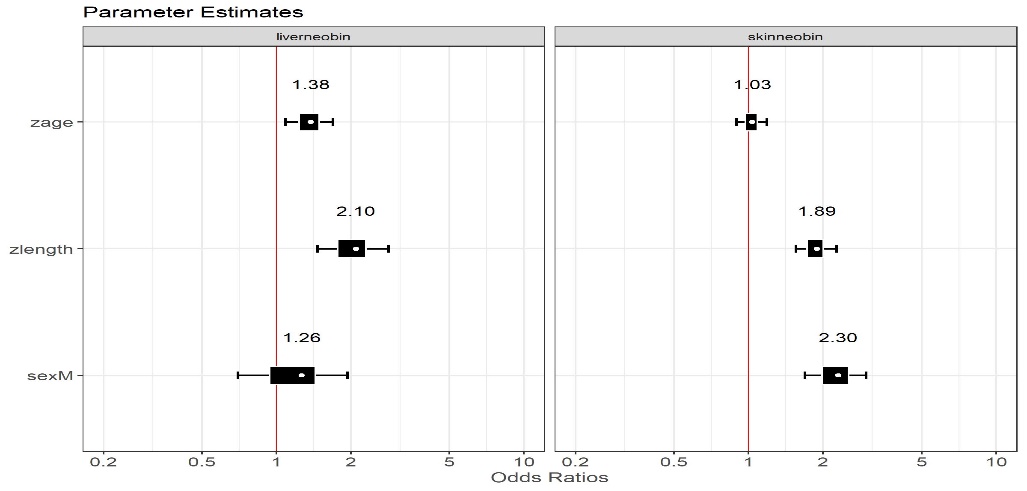


**A**


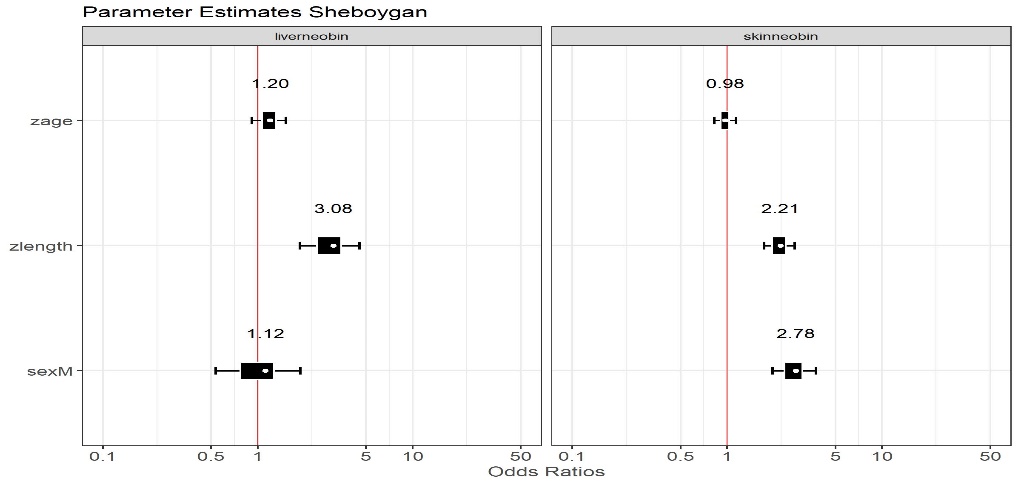


**B**.


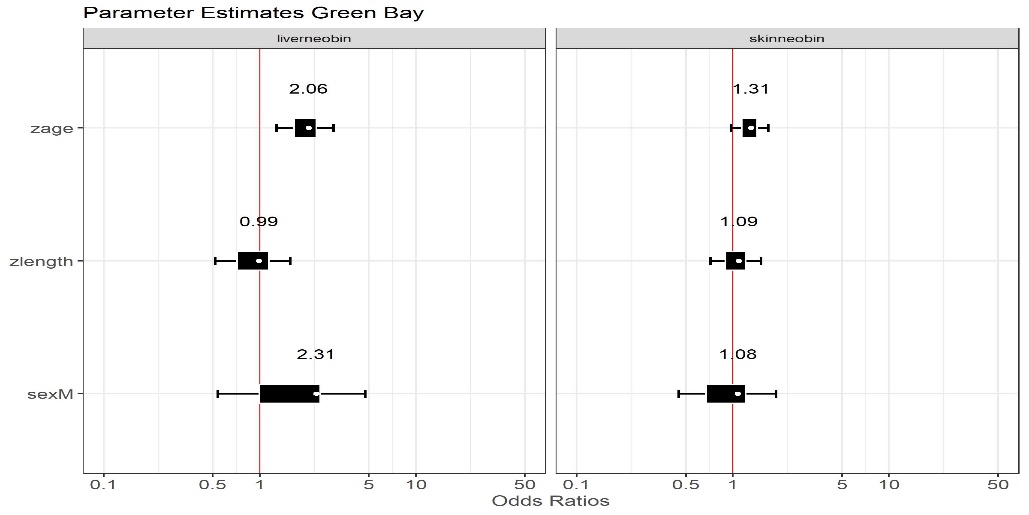


**C**.

**Supplemental Figure 2. Posterior mean and 95% credible intervals for site-level random effects (A) and year-level random effects (B) from Bayesian logistic regression models of neoplasia presence in skin (left panels) and liver (right panels).** Random effects are plotted on the log-odds scale and represent the deviation of each group (site or year) from the overall model intercept, after accounting for fixed effects (e.g., sex, length, age). Points near zero suggest little deviation from the overall mean neoplasia risk, while values above or below zero indicate relatively higher or lower risk, respectively.


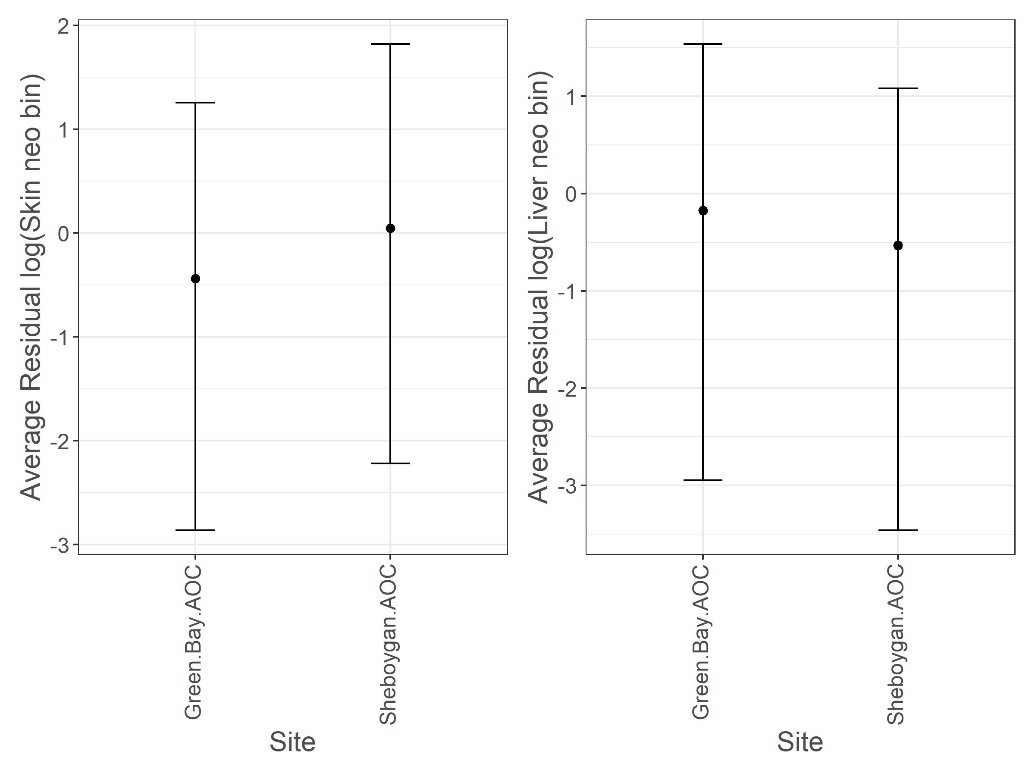


**A**


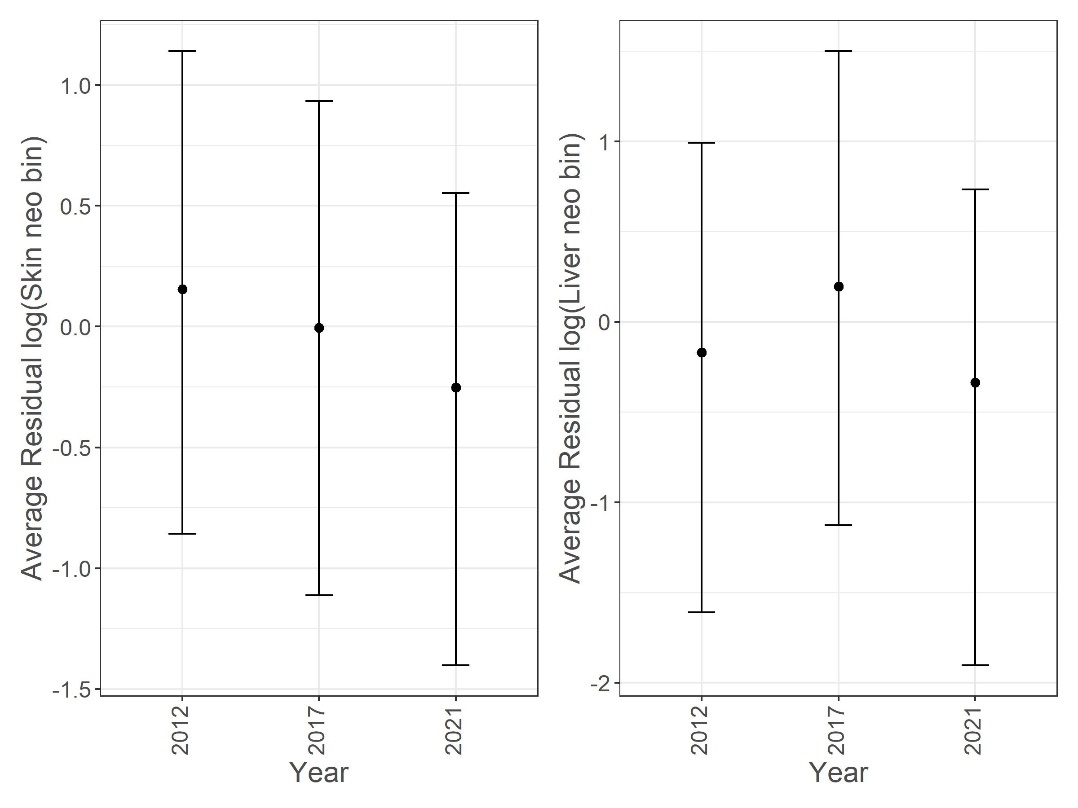


**B**
